# Size variation in mid-Holocene North Atlantic Puffins indicates a dynamic response to climate change

**DOI:** 10.1371/journal.pone.0246888

**Published:** 2021-02-24

**Authors:** Samuel James Walker, Hanneke Johanna Maria Meijer

**Affiliations:** 1 Department of Natural History, University Museum of Bergen, Bergen, Hordaland, Norway; 2 Human Origins Program, National Museum of Natural History, Smithsonian Institution, Washington, DC, United States of America; MARE – Marine and Environmental Sciences Centre, PORTUGAL

## Abstract

Seabirds are one of the most at-risk groups, with many species in decline. In Scandinavia, seabirds are at a heightened risk of extinction due to accelerated global warming. Norway is home to significant portion of the European Atlantic Puffin (*Fratercula arctica*) populations, but Norwegian populations have declined significantly during the last decades. In this paper we use biometric data from modern and archaeological *F*. *arctica* specimens to investigate patterns in body size variation over time of this iconic species. We aimed to set out a baseline for our archaeological comparison by firstly investigating whether modern subspecies of *F*. *arctica* are reflected in the osteological characters and are enough to distinguish subspecies from the bones alone. We then investigated if archaeological remains of *F*. *arctica* differ in size from the modern subspecies. Our results show that the subspecies *Fratercula arctica naumanni* was distinctly larger than the other subspecies. However, *Fratercula arctica arctica* and *Fratercula arctica grabae* were difficult to separate based on size. This generally supports ornithological observations. Post-Medieval *F*. *arctica* bones from Måsøy were similar to modern *F*. *a*. *arctica* populations. The mid-Holocene remains from Dollsteinhola overlaps with the modern size ranges of *F*. *a*. *arctica* and *F*. *a*. *grabae* but are generally shorter and more robust. Dollsteinhola is located close to the borders of the modern breeding ranges of both *F*. *a*. *arctica* and *F*. *a*. *grabae*. We consider it therefore likely that given the mid-Holocene climatic oscillations, breeding ranges of the two subspecies shifted north or south accordingly. However, this does not explain the different proportions of the Dollsteinhola specimens. Our data provide the first evidence for shifting distributions in ancient Atlantic Puffins and represent the first osteological analysis of *Fratercula arctica* subspecies.

## Introduction

Seabirds are one of the most at-risk bird groups with approximately half of all seabirds species in decline [1–3], and 110 species (31%) regarded as threatened by the IUCN red list. Seabirds in Scandinavia are at the limits of their distribution, and at a heightened risk of extinction due to further warming in northern biomes as a result of Arctic amplification [4]. Monitoring of seabird communities along the Norwegian coast [5] found declining populations across all major ecological groups, with several species, including the iconic Atlantic Puffin (*Fratercula arctica* [6]), having declined more than 50% in the last 25 years.

The Atlantic Puffin is an iconic species, recognised for its distinct appearance, and one of the six Alcidae species breeding in Norway. The vast majority (c.80%) of the European Atlantic Puffin population is found in Iceland and Norway. In addition, Norwegian *F*. *arctica* constitute 25–30% of the global population [7]. Monitoring studies [5] indicate that the once large populations of Atlantic Puffin in the Norwegian Sea have been significantly reduced from 1.6 million pairs in 1980 to 600 000 at present, likely as a result of environmental and anthropogenic changes.

To understand the responses of modern species to environmental change, it is crucial to understand the responses of ancient species to past environmental perturbations. Organisms may adapt to environmental oscillations by changes in body size, which in turn is linked to a number of life history traits. Only a handful of studies have looked at body size variation of Scandinavian bird species through time. For the Common Eider (*Somateria mollissima*), Ericson [8] found that there was stasis in body size between 7000 BCE and 1000 CE, with a subsequent decrease in average size between 1000–1900 CE. This decrease was likely linked to changes in levels of food competition [8]. A study by Hufthammer [9] on the extinct Great Auk (*Pinguinus impennis*) showed that lower limb bones were larger in the past, a pattern particularly evident in material older than 5000 years BP and attributed to changes in climatic conditions.

There are three recognised subspecies of the Atlantic Puffin. The nominate subspecies *Fratercula arctica arctica* breeds in Iceland, Norway (from the Runde colony northwards [10]), Bear Island, southern Novaya Zemlya, south-west Greenland and eastern North America. *Fratercula arctica naumanni* [11] is geographically distributed above the Arctic Circle in north-west and eastern Greenland, Spitsbergen and northern Novaya Zemlya. *Fratercula arctica grabae* [12] occupies more southern climes and occurs in Britain, Ireland, Faroes, Channel Islands, France and Norway (from Utvær southwards [10]) [13,14]. Ornithological studies have sought to distinguish between the three subspecies through weight, bill length, bill depth and wing length [15–20]. These studies have found that subspecies can be loosely separated on size alone. *Fratercula arctica naumanni* is the largest, with *F*. *a*. *grabae* being the smallest, and *F*. *a*. *arctica* falling in between. Despite the differences, there is considerable overlap between the subspecies, especially between the nominate *F*. *a*. *arctica* and *F*. *a*. *grabae*. Protein studies [21] show low genetic variation between *F*. *a*. *arctica* and *F*. *a*. *grabae*, and has led many to question the validity of Atlantic Puffin subspecies [22,23]. A recent study on population structure of the Atlantic Puffin using whole genome data [24] identified four population clusters that disagrees with the traditional view of three subspecies. This suggests that Atlantic Puffin taxonomy is more complicated than previously assumed.

Studies based on body weights and external measurements have given an important insight into size differences between populations of *F*. *arctica*. These studies have identified a north-south clinal pattern [16,25] with body size increasing at higher latitudes, which is likely influenced by environmental factors, like ocean temperature and food quality [21,22]. It would therefore be expected that at times when environmental conditions differed from the present day, we would see differences in the body size of *F*. *arctica* populations.

**Fig 1:**
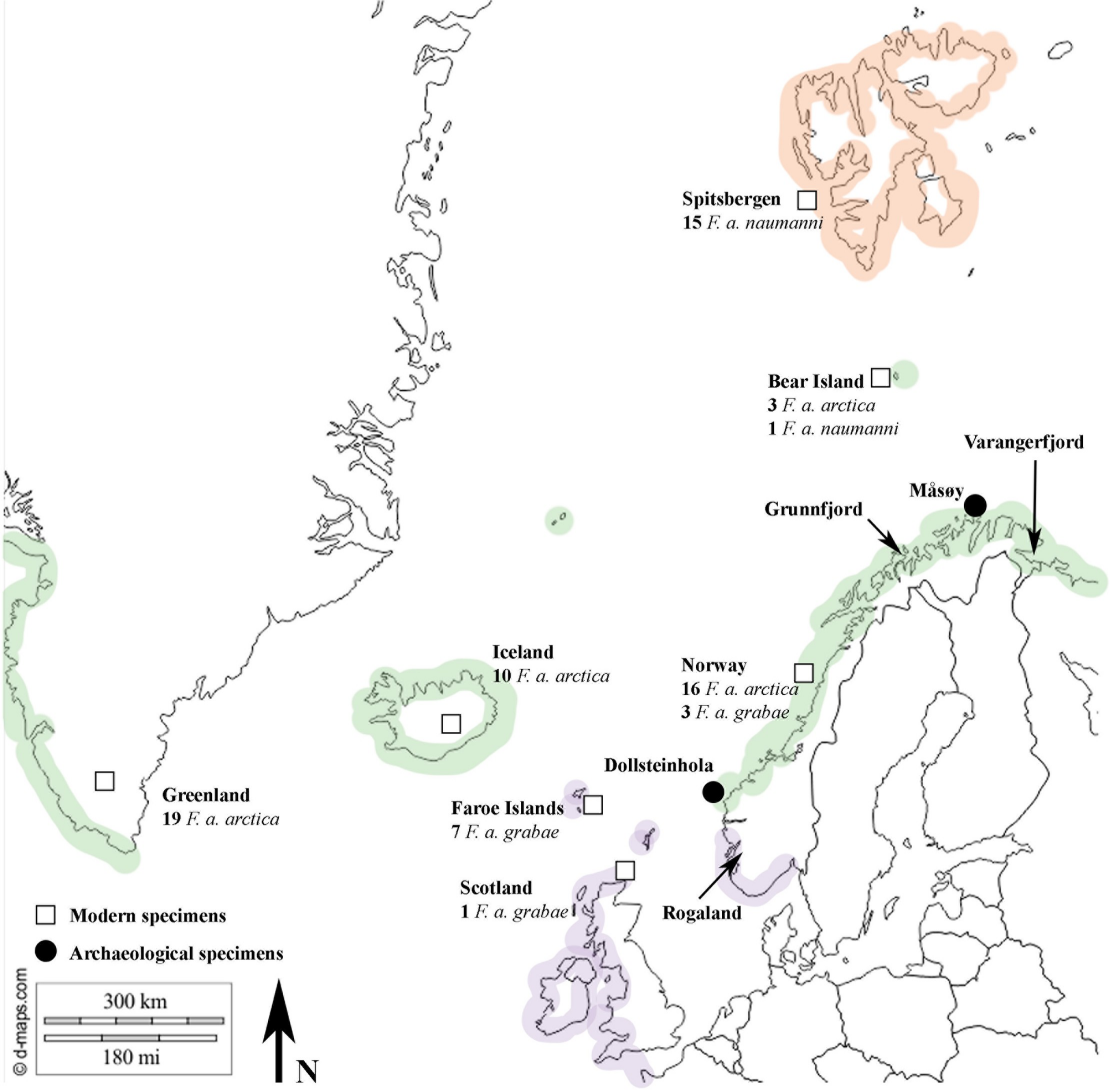
Map of archaeological sites (Dollsteinhola and Måsøy) and the locations of the comparative *Fratercula arctica* specimens. The sites investigated by Olsen [26] and Lahtiperä [27] are also indicated on the map. The known breeding distribution of all three subspecies is clearly indicated on the map (purple; *F*. *a*. *grabae*, green; *F*. *a*. *arctica* and orange; *F*. *a*. *naumanni*), data on breeding distributions is taken from Dementev and Gladkov [28]. Reprinted from d-maps [29] under a CC BY licence, with permission from [d-maps], original copyright [2020].

Although *Fratercula arctica* is regularly found in the archaeological record of coastal Norwegian sites (predominantly north of Bergen), there are few osteological studies on their remains. Olsen’s [26] work on Neolithic sites along the Varangerfjord in northern Norway (See Fig 1) is the main piece of osteological work on *F*. *arctica*. The study found that *F*. *a*. *arctica* was the only subspecies present on the site and that it was slightly larger than modern *F*. *a*. *arctica* populations. This led to the conclusion that conditions in the Varangerfjord were colder during the Stone Age. A similar study by Lahtiperä [27] found that Atlantic Puffin remains from Grunnfjord farm, a 16^th^–18^th^ Century site in northern Norway (See Fig 1), displayed a homogenous population of *F*. *a*. *arctica*. Unlike Olsen’s [26] study, the material from Grunnfjord Farm was regarded as the same size as modern *F*. *a*. *arctica* populations. To further explore past and present variation in Atlantic Puffin body size and its link to environmental conditions, we measured skeletal specimens of modern *F*. *arctica* and archaeological *F*. *arctica* remains from two Norwegian sites. Specifically, we aimed 1) to investigate whether modern subspecies of *F*. *arctica* (from ornithological observations) are reflected in the osteological characters, and are enough to distinguish subspecies from the bones alone, and 2) to determine if archaeological remains of *F*. *arctica* differ in size from the modern populations in relation to climatic change and other factors.

## Methods

### Modern comparative material

Modern puffin specimens examined for this study are part of the Osteological collections at the Bergen Natural History Museum and were inspected on site by SJW and HJMM. Additional specimens at the Natural History Museum of Denmark were examined by SJW, while specimens at Natural History Museum of Geneva and the Natural History Museum at Tring were examined by A. Cibois and J. White, respectively. All specimens, with the exception of 3, were measured by SJW. We measured seven skeletal elements (coracoid, humerus, ulna, carpometacarpus, femur, tibiotarsus and tarsometatarsus) from 75 modern Atlantic Puffin specimens using digital calipers. Measurements followed the conventions set out in Von den Driesch [30]. Additional measurements were taken from Kraft [31] (See **S1 Text** for details on how measurements were taken). In order to compare size of the bones between the 3 subspecies of *F*. *arctica*, it was integral to select modern specimens confidently identified to subspecies. We therefore only used specimens that had been identified to subspecies upon collection (often on diagnostic external characters such as living weights, bill length, bill depth and wing length), and the location and time of year of collection of the specimen were cross-checked with the subspecies breeding range. Specimens from the Faroe Islands that were labelled as *F*. *a*. *arctica* were considered as *F*. *a*. *grabae* in this study (Museum numbers; NHMD 223250, NHMD 223251, NHMD 223252, NHMD 223258, NHMD 223263). In addition, a specimen of *F*. *a*. *arctica* from Bore, Rogaland (Fig 1) was also considered as *F*. *a*. *grabae* (B 3052). Finally, a specimen labelled as *F*. *a*. *arctica* from Spitsbergen was regarded as *F*. *a*. *naumanni* (BM 10341). These seven specimens (NHMD 223250, NHMD 223251, NHMD 223252, NHMD 223258, NHMD 223263, B 3052 and BM 10341) were changed due to their geographic origin during the breeding season, all were found outside of the nominate breeding range and were therefore highly likely to belong to one the subspecies.

Any subspecies specimens that were collected outside their temporal and geographic breeding range (breeding colonies occupied between late March—Mid August/early September [22]) were not included in this study. Forty-four specimens fell into this category and were not included. This prevents uncertainty over the inclusion of migratory birds, as the migration patterns of Atlantic Puffin cause mixed colonies of the subspecies during the winter months. Through our subspecies cross-check we were able to use 48 specimens of the nominate *Fratercula arctica arctica*, 11 specimens of subspecies *Fratercula arctica grabae* and 16 of subspecies *Fratercula arctica naumanni*. These 75 specimens have been measured from the natural history collections held at the University Museum of Bergen, the Natural History Museum of Denmark, the Natural History Museum of Geneva and the Natural History Museum at Tring (See S1 Appendix). Both complete and partial specimens were included within this study, covering the majority of the Atlantic Puffins breeding range. The specimens were collected from Norway and Spitsbergen (n = 38), Greenland (n = 19), Iceland (n = 10), Faroe Islands (n = 7) and Scotland (n = 1) (See Fig 1). In order to be thorough and to check for any effects of sexual dimorphism, sex was recorded for all modern specimens. We found that males were on average between 0.3–2.8% larger than females. However, there was also a large overlap between the sexes. Given this and the relatively low percentage of sexual dimorphism, we decided to group males and females for comparisons with archaeological material, which also likely represents a mix of males and females.

### Archaeological material

Morphologically *Fratercula arctica* can be separated from other closely related Alcidae species based on a number of characteristics and the use of an extensive comparative collection. Morphological characters specific to *F*. *arctica*, such as a distinct facies articularis sternalis of the coracoid, and the tarsometatarsus being much shorter and more robust than some of the similar sized Alcids were utilised in this study (Additional characters and comparisons with other Auks can be found in Olsen [26]). Certain skeletal elements, such as the coracoid, humerus, tibiotarsus and tarsometatarsus, are more diagnostic than others. All archaeological material was reidentified using the extensive modern comparative collections held at the University Museum of Bergen. We made sure to use only specimens that could be confidently assigned to *Fratercula arctica*. In total 380 archaeological specimens were included within this study, 227 from the site of Dollsteinhola and 153 from Måsøy.

The cave site of Dollsteinhola (stored under number JS 706 in the University Museum of Bergens collections) is located on the west coast of Norway on Sandøya Island in the county Møre and Romsdal at a latitude of 62°N (See Fig 1). Over 70,000 bones were recovered from the site, representing 124 bird species [32]. Dollsteinhola has a wide date range of between c.6600 – 3600 BP [32]. This date range encompasses a short period of the late Mesolithic and into the Neolithic, and there is also some evidence of Bronze Age material at the site. The older material at this site is non-anthropogenic and represents a natural deposited assemblage, however, by the Bronze Age it is an anthropogenic assemblage. In terms of climatic change, Dollsteinhola is of great interest, representing a mid-Holocene (7.3–4.8 BP) assemblage, a period where higher summer temperatures were around 1.5–2.0°C warmer than present [33–37]. According to the temperature curve for the west coast of Norway [37] the post-glacial warm period lasted until 4000 years BP. The younger material from Dollsteinhola dates to the late-Holocene (4.8 BP–present), a period of decreasing summer temperatures [33–35] and higher levels of precipitation [38]. This indicates that some of the later specimens from this site would have been from a colder and wetter period.

Måsøy (stored under number JS 673 in the University Museum of Bergen’s collections) is located in the county of Troms and Finnmark in the far north of Norway, at a latitude of 71°N (See Fig 1). The bone material from Måsøy is from a Post-Medieval midden and has been dated to 1620–1770 CE [39], representing an anthropogenic assemblage from the Little Ice Age (LIA). The Little Ice Age was a period of increased glacial activity in the late Holocene and temperature reconstructions reveal oscillating warm and cold periods across the Northern Hemisphere [40]. For northern Norway, the coldest period was reconstructed for the 17^th^ century [41].

#### 2.1 Data analysis

We first explored differences in size between groups using descriptive statistics. All data were tested for normality by looking at the variances and the Shapiro-Wilk test for normality. To statistically test for differences in size between the 3 modern subspecies, and between the modern subspecies and the archaeological material, we used one-way ANOVA’s. We considered p-values ≤ 0.05 statistically significant. All analyses were performed using the analytical program PAST 4.03 [42].

## Results

### The data

Data tables presenting the mean measurements in millimetres along with the variance and number of specimens for the coracoid, humerus, ulna, carpometacarpus, femur, tibiotarsus and tarsometatarsus are given in Tables 1–7.

**Table 1:** Coracoid data.

Taxon	No. specimens	Lm	Standard deviation	Bb	Standard deviation	BF	Standard deviation
*F*. *a*. *arctica*	48	35.40	1.36	12.78	0.94	10.01	0.67
*F*. *a*. *grabae*	10	34.57	1.94	12.55	0.57	9.51	0.51
*F*. *a*. *naumanni*	16	38.44 (n = 15)	0.67	13.86	0.73	10.95	0.64
Dollsteinhola	39	34.40 (n = 35)	1.59	13.64 (n = 1)	0	10.21 (n = 36)	1.11
Måsøy	28	35.47 (n = 24)	0.71	12.59 (n = 3)	0.23	9.82 (n = 26)	0.52

Mean measurements (in mm) and the variance for the coracoid of modern *F*. *a*. *arctica*, *F*. *a*. *grabae* and *F*. *a*. *naumanni*, and archaeological specimens from Dollsteinhola and Måsøy. Abbreviations: Lm, medial length, Bb, basal breadth, BF, breadth of the facies articularis sternalis. The number of specimens is presented in the second column, any deviations are indicated in brackets next to the mean values.

**Table 2:** Humerus data.

Taxon	No. specimens	GL	Standard deviation	Bp	Standard deviation	SC	Standard deviation	Bd	Standard deviation	KB	Standard deviation
*F*. *a*. *arctica*	48	65.21 (n = 45)	1.70	15.07	0.68	3.21 (n = 46)	0.17	6.95 (n = 44)	0.28	3.97 (n = 44)	0.20
*F*. *a*. *grabae*	11	62.74	3.03	14.18	0.76	3.14	0.29	6.76	0.41	3.85	0.41
*F*. *a*. *naumanni*	16	69.66 (n = 14)	1.71	16.00	0.50	3.46	0.16	7.37	0.25	4.29	0.21
Dollsteinhola	32	63.38 (n = 14)	2.11	14.54 (n = 25)	0.43	3.27 (n = 22)	0.26	6.92 (n = 19)	0.26	3.95 (n = 20)	0.22
Måsøy	37	68.96 (n = 1)	0	15.15 (n = 13)	0.80	3.33 (n = 7)	0.19	6.98 (n = 25)	0.33	4.05 (n = 25)	0.19

Mean measurements (in mm) and the variance for the humerus of modern *F*. *a*. *arctica* and subspecies *F*. *a*. *grabae* and *F*. *a*. *naumanni*, and archaeological specimens from Dollsteinhola and Måsøy. Abbreviations: GL, greatest length, Bp, breadth of the proximal end, SC, smallest breadth of the corpus, Bd, breadth of the distal end, KB, smallest depth of the distal shaft. The number of specimens is presented in the second column, any deviations are indicated in brackets next to the mean values.

**Table 3:** Ulna data.

Taxon	No. specimens	GL	Standard deviation	Dip	Standard deviation	Bp	Standard deviation	Tp	Standard deviation	SC	Standard deviation	Did (mm)	Standard deviation
*F*. *a*. *arctica*	27	50.76	1.19	8.15	0.39	6.84	0.33	6.17	0.27	2.96	0.13	7.45	0.32
*F*. *a*. *grabae*	6	50.92	2.56	8.77	0.71	6.57	0.16	6.78	0.73	3.26	0.60	7.47	0.26
*F*. *a*. *naumanni*	16	55.02 (n = 13)	1.25	8.82 (n = 12)	0.48	7.37 (n = 12)	0.24	6.71 (n = 12)	0.21	3.25	0.18	8.09	0.17
Dollsteinhola	18	49.46 (n = 17)	2.44	7.98	0.49	6.77	0.40	6.13	0.35	2.96 (n = 16)	0.29	7.33 (n = 17)	0.32
Måsøy	28	51.65 (n = 24)	0.97	8.20 (n = 26)	0.41	6.92 (n = 26)	0.30	6.30 (n = 25)	0.24	3.06 (n = 26)	0.14	7.53 (n = 26)	0.25

Mean measurements (in mm) and the variance for the ulna of modern *F*. *a*. *arctica* and subspecies *F*. *a*. *grabae* and *F*. *a*. *naumanni*, and archaeological specimens from Dollsteinhola and Måsøy. Abbreviations: GL, greatest length, Dip, diagonal of the proximal end, Bp, breadth of the proximal end, Tp, depth of the proximal end, SC, smallest breadth of the corpus, Did, diagonal of the distal end. The number of specimens is presented in the second column, any deviations are indicated in brackets next to the mean values.

**Table 4:** Carpometacarpus data.

Taxon	No. specimens	GL	Standard deviation	Bp	Standard deviation	Did	Standard deviation	HS	Standard deviation
*F*. *a*. *arctica*	27	34.15	0.90	8.02	0.42	5.04	0.29	3.89	0.23
*F*. *a*. *grabae*	6	34.26	1.60	8.30	0.43	5.01	0.18	4.12	0.58
*F*. *a*. *naumanni*	16	37.12	0.81	8.64	0.33	5.54	0.34	4.18	0.24
Dollsteinhola	27	32.84 (n = 25)	1.42	8.00	0.39	5.31 (n = 25)	0.38	3.67 (n = 24)	0.40
Måsøy	23	34.85 (n = 21)	0.77	8.17 (n = 22)	0.30	5.21 (n = 22)	0.34	3.89 (n = 22)	0.22

Mean measurements (in mm) and the variance for the carpometacarpus of modern *F*. *a*. *arctica* and subspecies *F*. *a*. *grabae* and *F*. *a*. *naumanni*, and archaeological specimens have also been included from Dollsteinhola and Måsøy. Abbreviations: GL, greatest length, Bp, breadth of the proximal end, Did, diagonal of the distal end, HS, height of the symphysis. The number of specimens is presented in the second column, any deviations are indicated in brackets next to the mean values.

**Table 5:** Femur data.

Taxon	No. specimens	GL	Standard deviation	Bp	Standard deviation	Dp	Standard deviation	SC	Standard deviation	Bd	Standard deviation	Dd	Standard deviation
*F*. *a*. *arctica*	47	39.66	1.56	7.88	0.41	5.24	0.30	2.96 (n = 46)	0.17	7.12	0.38	6.12 (n = 46)	0.38
*F*. *a*. *grabae*	8	38.27	2.41	7.32	0.46	5.18	0.29	2.87	0.14	6.80	0.44	5.60	0.60
*F*. *a*. *naumanni*	16	41.97	1.14	8.37	0.38	5.63	0.26	3.15	0.18	7.53	0.29	6.55	0.23
Dollsteinhola	12	37.46	1.25	7.63	0.40	4.92	0.25	2.98	0.16	7.05	0.40	5.95	0.33
Måsøy	14	39.76 (n = 9)	0.85	7.70 (n = 10)	0.41	5.12 (n = 9)	0.30	3.08	0.17	7.16 (n = 11)	0.26	6.13 (n = 11)	0.19

Mean measurements (in mm) and the variance for the femur of modern *F*. *a*. *arctica* and subspecies *F*. *a*. *grabae* and *F*. *a*. *naumanni*, archaeological specimens from Dollsteinhola and Måsøy. Abbreviations: GL, greatest length, Bp, breadth of the proximal end, Dp, depth of the proximal end, SC, smallest breadth of the corpus, Bd, breadth of the distal end, Dd, depth of the distal end. The number of specimens is presented in the second column, any deviations are indicated in brackets next to the mean values.

**Table 6:** Tibiotarsus data.

Taxon	No. specimens	La	Standard deviation	Dip	Standard deviation	Bp	Standard deviation	SC	Standard deviation	Bd	Standard deviation	Dd	Standard deviation
*F*. *a*. *arctica*	27	62.90	1.56	8.45	0.41	5.84 (n = 26)	0.35	3.22	0.19	5.73	0.27	6.02	0.28
*F*. *a*. *grabae*	6	63.55	3.66	9.11 (n = 5)	0.50	5.69 (n = 4)	0.44	3.14	0.32	5.74	0.31	5.60	1.15
*F*. *a*. *naumanni*	16	68.29	1.98	9.10	0.27	6.47 (n = 15)	0.22	3.48	0.25	6.28	0.26	6.37	0.23
Dollsteinhola	15	61.37 (n = 8)	1.89	8.43 (n = 12)	0.31	5.88 (n = 12)	0.20	3.25	0.21	6.04 (n = 11)	0.38	5.85 (n = 11)	0.24
Måsøy	15	63.34 (n = 2)	1.34	8.6 (n = 11)	0.31	5.99 (n = 11)	0.36	3.41 (n = 12)	0.21	5.93 (n = 5)	0.29	5.93 (n = 5)	0.29

Mean measurements (in mm) and the variance for the tibiotarsus of modern *F*. *a*. *arctica* and subspecies *F*. *a*. *grabae* and *F*. *a*. *naumanni*, and archaeological specimens from Dollsteinhola and Måsøy. Abbreviations: La, axial length (defined as, from the tuberculum centrale to the distal border of the trochlea tibiotarsi), Dip, diagonal of the proximal end (defined as, from the condylus medialis femoralis to the crista lateralis), Bp, breadth of the proximal end, SC, smallest breadth of the corpus, Bd, breadth of the distal end, Dd, depth of the distal end. The number of specimens is presented in the second column, any deviations are indicated in brackets next to the mean values.

**Table 7:** Tarsometatarsus data.

Taxon	No. specimens	GL	Standard deviation	Bp	Standard deviation	SC	Standard deviation	Bd	Standard deviation
*F*. *a*. *arctica*	24	27.84 (n = 23)	0.97	6.43	0.32	3.57 (n = 23)	0.30	6.96 (n = 21)	0.28
*F*. *a*. *grabae*	3	29.71	1.85	6.24 (n = 2)	0.23	3.49 (n = 2)	0.01	6.83 (n = 2)	0.24
*F*. *a*. *naumanni*	15	30.03	1.23	7.09	0.31	3.86	0.19	7.43	0.27
Dollsteinhola	84	27.21 (n = 70)	1.77	6.22 (n = 72)	0.36	3.57 (n = 83)	0.29	6.74 (n = 69)	0.37
Måsøy	8	28.51 (n = 6)	0.79	6.56 (n = 6)	0.27	3.63 (n = 7)	0.19	7.14	0.22

Mean measurements (in mm) and the variance for the tarsometatarsus of modern *F*. *a*. *arctica* and subspecies *F*. *a*. *grabae* and *F*. *a*. *naumanni*, and archaeological specimens from Dollsteinhola and Måsøy. Abbreviations: GL, greatest length, Bp, breadth of the proximal end, SC, smallest breadth of the corpus, Bd, breadth of the distal end. The number of specimens is presented in the second column, any deviations are indicated in brackets next to the mean values.

### Size differences between subspecies

The results of the one-way ANOVA (Table 8) show that on the whole comparisons between *F*. *a*. *arctica* and *F*. *a*. *grabae* were not statistically significant. However, the greatest length of the humerus (Table 8B) and the greatest length of the tarsometatarsus (Table 8G) did identify a significant difference between them, along with a number of other measurements (See Table 8). The comparisons between *F*. *a*. *grabae* and *F*. *a*. *naumanni* (Table 8) show that almost all measurements are statistically different. A small number of measurements showed no difference (Table 8C, 8D, 8F and 8G). Finally, comparisons between *F*. *a*. *arctica* and *F*. *a*. *naumanni* show that all measurements are statistically significant.

**Table 8:** P-values for the one-way ANOVA for modern subspecies.

**A**	Coracoid ANOVA results																								
	***F*. *a*. *arctica***													***F*. *a*. *naumanni***											
	Lm					Bb				BF				Lm				Bb				BF			
***F*. *a*. *grabae***	0.185					0.721				0.071				< .001				< .001				< .001			
***F*. *a*. *arctica***														< .001				< .001				< .001			
**B**	Humerus ANOVA results																								
	***F*. *a*. *arctica***													***F*. *a*. *naumanni***											
	GL		Bp				SC		Bd			KB		GL		Bp			SC		Bd			KB	
***F*. *a*. *grabae***	< .001		< .001				0.554		0.133			0.292		< .001		< .001			< .001		< .001			< .001	
***F*. *a*. *arctica***														< .001		< .001			< .001		< .001			< .001	
**C**	Ulna ANOVA results																								
	***F*. *a*. *arctica***													***F*. *a*. *naumanni***											
	GL	Dip				Bp		Tp		SC			Did	GL	Dip			Bp		Tp		SC			Did
***F*. *a*. *grabae***	0.969	0.014				0.128		< .001		0.026			0.991	< .001	0.970			< .001		0.904		1			< .001
***F*. *a*. *arctica***														< .001	< .001			< .001		< .001		< .001			< .001
**D**	Carpometacarpus ANOVA results																								
	***F*. *a*. *arctica***													***F*. *a*. *naumanni***											
	GL			Bp				Did			HS			GL			Bp			Did			HS		
***F*. *a*. *grabae***	0.963			0.258				0.961			0.218			< .001			0.184			< .001			0.893		
***F*. *a*. *arctica***														< .001			< .001			< .001			0.009		
**E**	Femur ANOVA results																								
	***F*. *a*. *arctica***													***F*. *a*. *naumanni***											
	GL	Bp				Dp		SC		Bd			Dd	GL	Bp			Dp		SC		Bd			Dd
***F*. *a*. *grabae***	0.064	0.002				0.832		0.329		0.073			0.002	< .001	< .001			< .001		< .001		< .001			< .001
***F*. *a*. *arctica***														< .001	< .001			< .001		< .001		< .001			< .001
**F**	Tibiotarsus ANOVA results																								
	***F*. *a*. *arctica***													***F*. *a*. *naumanni***											
	La	Dip				Bp		SC		Bd			Dd	La	Dip			Bp		SC		Bd			Dd
***F*. *a*. *grabae***	0.756	0.002				0.644		0.759		1			0.108	< .001	0.997			< .001		0.009		< .001			0.002
***F*. *a*. *arctica***														< .001	< .001			< .001		0.002		< .001			0.046
**G**	Tarsometatarsus ANOVA results																								
	***F*. *a*. *arctica***													***F*. *a*. *naumanni***											
	GL				Bp			SC			Bd			GL			Bp			SC			Bd		
***F*. *a*. *grabae***	0.028				0.700			0.898			0.788			0.897			0.003			0.152			0.015		
***F*. *a*. *arctica***														< .001			< .001			0.005			< .001		

Summary of the p values from the one-way ANOVA results for all 7 elements investigated in this paper of modern *F*. *a*. *arctica*, *F*. *a*. *grabae* and *F*. *a*. *naumanni*. All p-values below 0.05 are regarded as statistically significant. For the full ANOVA results and details of normality testing see supplementary file (**S2 Appendix**).

### Size differences between modern and archaeological material

Comparisons of the modern subspecies with the archaeological specimens from Dollsteinhola and Måsøy, and between the two archaeological populations are presented in Table 9. The ANOVA results show that the subspecies *F*. *a*. *naumanni* is significantly different to the archaeological specimens at Dollsteinhola and Måsøy (Table 9). The comparison between the nominate *F*. *a*. *arctica* and Dollsteinhola shows that the length measurements of the coracoid, humerus, carpometacarpus and femur were all significantly different (Table 9A, 9B, 9D and 9E). Comparisons between *F*. *a*. *arctica* and Måsøy showed that they were not statistically different in any measurement. The subspecies *F*. *a*. *grabae* showed few statistical differences when compared to the Dollsteinhola population, the carpometacarpus was the most notable difference (Table 9D). Similarly, there were very few differences detected between *F*. *a*. *grabae* and the Måsøy specimens. Finally, comparisons between the two archaeological sites indicated significant differences in the greatest length of the upper limb bones (Table 9A, 9C and 9D) and the femur (Table 9E). Other measurements tended not to be significantly different.

**Table 9:** P-values for the one-way ANOVA for modern subspecies compared to archaeological populations.

**A**	Coracoid ANOVA results																								
	**Dollsteinhola**													**Måsøy**											
	Lm					Bb				BF				Lm				Bb				BF			
***F*. *a*. *grabae***	0.997									0.092				0.374				1				0.820			
***F*. *a*. *arctica***	0.008									0.774				1				0.981				0.851			
***F*. *a*. *naumanni***	< .001									0.017				< .001				0.088				< .001			
**Dollsteinhola**														0.023								0.297			
**B**	Humerus ANOVA results																								
	**Dollsteinhola**													**Måsøy**											
	GL		Bp				SC		Bd			KB		GL		Bp			SC		Bd			KB	
***F*. *a*. *grabae***	0.858		0.522				0.413		0.612			0.788				0.233			0.329		0.233			0.111	
***F*. *a*. *arctica***	0.018		0.009				0.734		0.994			0.993				0.994			0.594		0.994			0.642	
***F*. *a*. *naumanni***	< .001		< .001				0.058		< .001			< .001				< .001			0.641		< .001			0.015	
**Dollsteinhola**																0.955			0.971		0.955			0.544	
**C**	Ulna ANOVA results																								
	**Dollsteinhola**													**Måsøy**											
	GL	Dip				Bp		Tp		SC			Did	GL	Dip			Bp		Tp		SC			Did
***F*. *a*. *grabae***	0.307	0.004				0.706		< .001		0.064			0.838	0.849	0.055			0.130		0.012		0.335			0.984
***F*. *a*. *arctica***	0.070	0.744				0.951		0.988		1			0.627	0.278	0.995			0.880		0.609		0.508			0.816
***F*. *a*. *naumanni***	< .001	< .001				< .001		< .001		0.005			< .001	< .001	0.002			< .001		0.005		0.072			< .001
**Dollsteinhola**														< .001	0.528			0.529		0.401		0.649			0.141
**D**	Carpometacarpus ANOVA results																								
	**Dollsteinhola**													**Måsøy**											
	GL			Bp				Did			HS			GL			Bp			Did			HS		
***F*. *a*. *grabae***	0.035			0.390				0.279			0.016			0.762			0.940			0.646			0.848		
***F*. *a*. *arctica***	< .001			1				0.040			0.076			0.172			0.618			0.365			1		
***F*. *a*. *naumanni***	< .001			< .001				0.174			< .001			< .001			0.002			0.027			0.037		
**Dollsteinhola**														< .001			0.518			0.884			0.126		
**E**	Femur ANOVA results																								
	**Dollsteinhola**													**Måsøy**											
	GL	Bp				Dp		SC		Bd			Dd	GL	Bp			Dp		SC		Bd			Dd
***F*. *a*. *grabae***	0.756	0.468				0.282		0.574		0.573			0.218	0.254	0.309			0.996		0.041		0.236			0.017
***F*. *a*. *arctica***	< .001	0.339				0.007		0.996		0.976			0.575	1	0.699			0.795		0.146		0.998			1
***F*. *a*. *naumanni***	< .001	< .001				< .001		0.086		0.008			< .001	0.005	< .001			< .001		0.827		0.079			0.030
**Dollsteinhola**														0.007	0.996			0.479		0.564		0.958			0.744
**F**	Tibiotarsus ANOVA results																								
	**Dollsteinhola**													**Måsøy**											
	La	Dip				Bp		SC		Bd			Dd	La	Dip			Bp		SC		Bd			Dd
***F*. *a*. *grabae***	0.269	0.005				0.815		0.862		0.240			0.755	1	0.069			0.465		0.118		0.819			0.669
***F*. *a*. *arctica***	0.330	1				0.996		0.991		0.032			0.777	0.998	0.745			0.684		0.089		0.655			0.993
***F*. *a*. *naumanni***	< .001	< .001				< .001		0.038		0.265			0.017	0.014	0.006			0.002		0.933		0.145			0.253
**Dollsteinhola**														0.726	0.777			0.921		0.317		0.942			0.995
**G**	Tarsometatarsus ANOVA results																								
	**Dollsteinhola**													**Måsøy**											
	GL				Bp			SC			Bd			GL			Bp			SC			Bd		
***F*. *a*. *grabae***	0.054				1			0.993			0.995			0.810			0.782			0.970			0.767		
***F*. *a*. *arctica***	0.431				0.084			1			0.059			0.880			0.914			0.990			0.707		
***F*. *a*. *naumanni***	< .001				< .001			0.003			< .001			0.260			0.016			0.371			0.269		
**Dollsteinhola**														0.282			0.141			0.986			0.014		

Summary of the p-values for one-way ANOVA results for all 7 elements investigated in this paper. Comparison of modern *F*. *a*. *arctica*, *F*. *a*. *grabae* and *F*. *a*. *naumanni* with the archaeological material from Dollsteinhola and Måsøy. All p-values below 0.05 are regarded as statistically significant. For the full ANOVA results and details of normality testing see supplementary file (**S3 Appendix**).

## Discussion

### Subspecies

**Fig 2:**
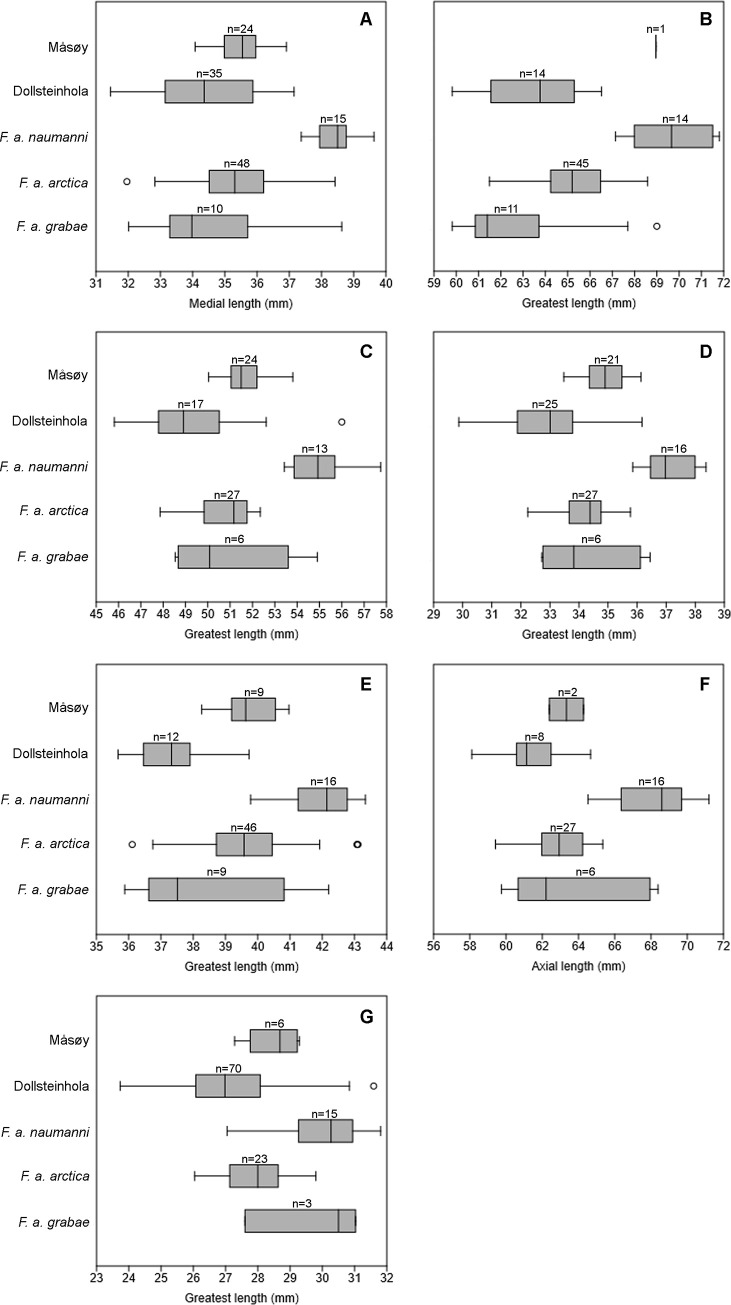
Boxplots of length measurements in modern and archaeological Atlantic Puffins. **A**, medial length of the coracoid. **B**, greatest length of the humerus. **C**, greatest length of the ulna. **D**, greatest length of the carpometacarpus. **E**, greatest length of the femur. **F**, axial length of the tibiotarsus. **G**, greatest length of the tarsometatarsus. Number of specimens included are represented above each boxplot. Outliers are indicated by circles beyond the standard error.

Our results have identified that there are osteological differences between the subspecies of Atlantic Puffin. It is evident that *F*. *a*. *naumanni* differs from *F*. *a*. *arctica* and *F*. *a*. *grabae* (Table 8; Fig 2); *F*. *a*. *naumanni* is larger than all other subspecies. This difference is not just detected in the greatest length but also in the dimensions of the proximal and distal end, suggesting that *F*. *a*. *naumanni* on the whole is proportionally larger than the other two subspecies. Despite this, there is a degree of overlap between *F*. *a*. *naumanni* and the other subspecies. External measurements have already identified that *F*. *a*. *naumanni* are larger than the other two subspecies [43,44]. Our data show that the larger size of *F*. *a*. *naumanni* is also reflected in the osteological data and that for most bones it is possible to separate *F*. *a*. *naumanni* from the other subspecies. The results have shown that greatest length of long bones is the measurement showing the most variation between the subspecies. This appears most pronounced in the upper limb elements (coracoid, humerus, ulna and carpometacarpus) where we see less of an overlap. Our findings would suggest that measurements which exceed the mean of *F*. *a*. *naumanni* are highly likely to belong to this subspecies (See Tables 1–7).

According to external measurements of *F*. *a*. *grabae* and *F*. *a*. *naumanni* these two subspecies should be at opposite ends of the size spectrum. However, some of the proximal dimensions of the ulna, carpometacarpus and tibiotarsus are not significantly different from one another, in addition the greatest length of the tarsometatarsus also shows no difference (Table 8). In this case we believe this is down to a small sample size. The skeletal elements (coracoid, humerus and femur) for which a larger sample of *F*. *a*. *grabae* was available show a significant difference to *F*. *a*. *naumanni* (Table 8). There are two specimens of *F*. *a*. *grabae* (NHMUK S/1973.66.92 and NHMD 223207) which do not fit with the expected smaller size of the subspecies. Both specimens are old (they were collected in 1946 and 1923 respectively), and it is not clear why they were assigned to subspecies *F*. *a*. *grabae*. It is possible they were wrongly assigned to *F*. *a*. *grabae*. However, both specimens come from known *F*. *a*. *grabae* breeding colonies during the breeding season; NHMUK S/1973.66.92 from Scotland taken in early June and NHMD 223207 from the Faroe Islands taken in August. It is possible that NHMD 223207 represents an early *F*. *a*. *arctica* winter migrant [22]. In addition, the wing length measurement associated with this specimen (173 mm), falls within the upper range for male *F*. *a*. *grabae* in Scotland 140 – 174mm [20], but also firmly within the range for *F*. *a*. *arctica* [16,19]. Alternatively, they may just be exceptionally large *F*. *a*. *grabae*, and can add important insight into the potentially high size variation within the subspecies *F*. *a*. *grabae*.

There is a high degree of overlap in the size of *F*. *a*. *arctica* and *F*. *a*. *grabae* (Fig 2). Only a limited number of specimens of *F*. *a*. *grabae* were available to us, and a larger sample size could have revealed a greater difference between the two. However, as mentioned in the introduction, *F*. *a*. *grabae* is often not regarded as a valid subspecies [14,21–23] and considered a part of *F*. *a*. *arctica* instead. When the mean values are looked at, the general trend does show a smaller size of *F*. *a*. *grabae* compared to *F*. *a*. *arctica*. However, there is a large amount of variation in the *F*. *a*. *grabae* bones, with some overlapping slightly with *F*. *a*. *naumanni*. This could indicate a large variation with a few particularly large individuals (see above). Alternatively, since one of the largest *F*. *a*. *grabae* specimens (NHMUK S/1973.66.92) was not measured by SJW, inter-observer variation may have resulted in a slight difference in measurements.

### Archaeological Atlantic Puffin

The results show that the *F*. *arctica* specimens from Måsøy represent a homogenous population that fits best with *F*. *a*. *arctica* (Table 9). This is in line with the archaeological findings from nearby Varangerfjord and Grunnfjord [26,27], and indicates that only *F*. *a*. *arctica*. was present in Northern Norway during the Little Ice Age, and there was no southward shift in the range of *F*. *a*. *naumanni*.

The results of *F*. *arctica* from Dollsteinhola indicate a large amount of variation in size, more so than seen in the Måsøy assemblage. Variation in Dollsteinhola ranges from quite large individuals (still within the *F*. *a*. *arctica* size range) to some very small individuals (smaller than both *F*. *a*. *grabae* and *F*. *a*. *arctica*). However, there is no evidence to suggest that *F*. *a*. *naumanni* was present at Dollsteinhola; the measurements show no similarity to the larger *F*. *a*. *naumanni* (Table 9) thus making it unlikely that they would have ventured, in any great numbers, as far south as Dollsteinhola during the Holocene.

**Fig 3:**
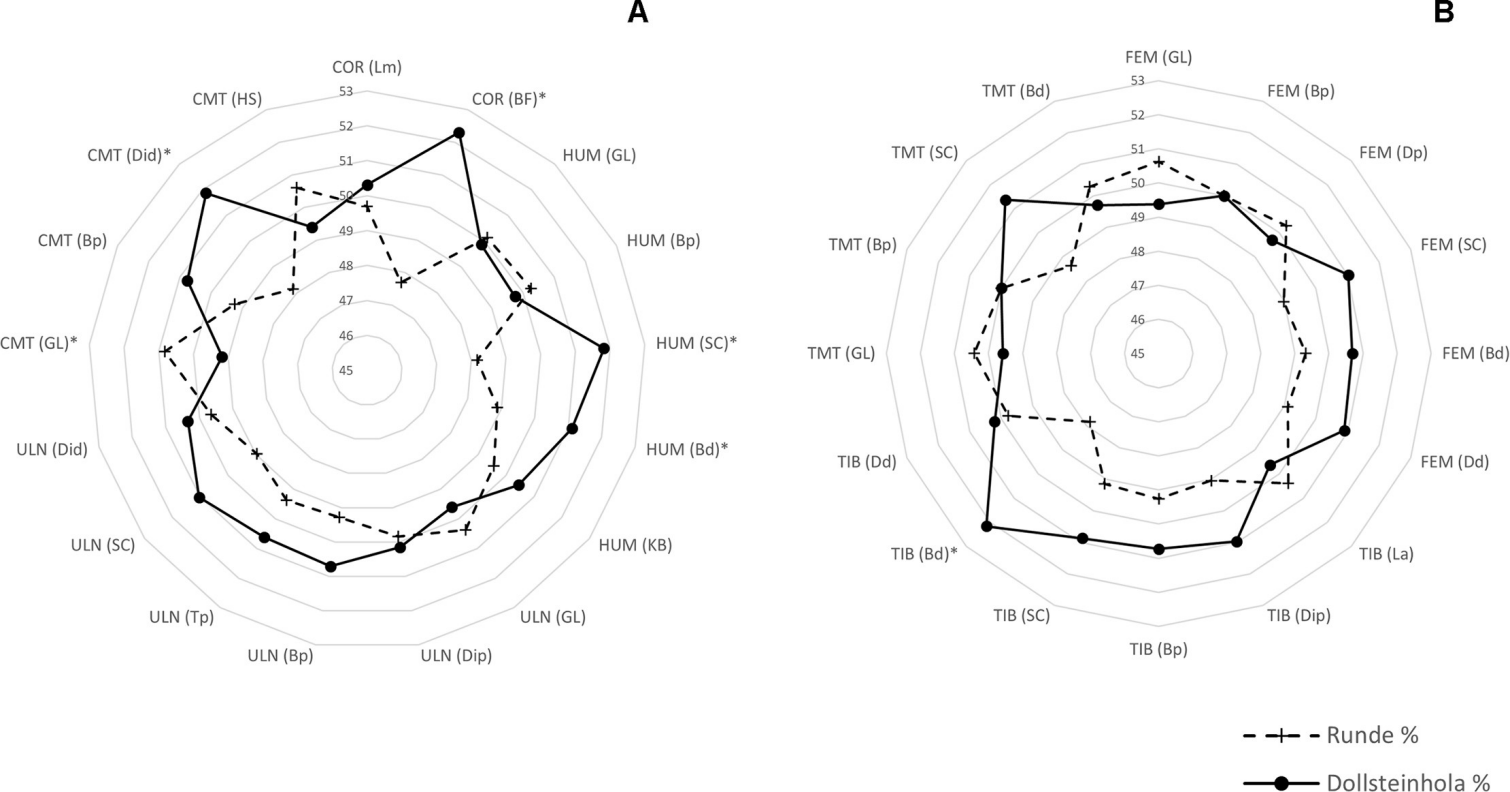
A comparison of the mean values for modern *F*. *a*. *arctica* from Runde and the *F*. *arctica* specimens from Dollsteinhola, taken as percentages to highlight which measurements are larger for the two groups. **A** represents measurements of the upper limbs, **B** represents measurements of the lower limbs. Measurements with * are statistically significant. Abbreviations of the bone elements are as follows; COR = coracoid, HUM = humerus, ULN = ulna, CMT = carpometacarpus, FEM = femur, TIB = tibiotarsus, TMT = tarsometatarsus. The mean values can be found in the supplementary material (**S1 Table**).

The Dollsteinhola specimens overlap in size with both *F*. *a*. *grabae* and smaller *F*. *a*. *arctica* specimens, but there are differences in individual elements. The comparison between Dollsteinhola and *F*. *a*. *arctica* show significant differences in the coracoid, humerus, carpometacarpus and femur (Table 9). The mean length of the Dollsteinhola specimens (Fig 2) is consistently smaller than current *F*. *a*. *arctica* populations, albeit not statistically significant (Table 9). The geographically closest modern population to Dollsteinhola is located just 20 Km to the north at Runde. Runde is home to the largest Norwegian *F*. *a*. *arctica* colony south of the Arctic Circle [22] (estimated at 50–70,000 breeding pairs in 2014 [45]). A direct comparison to 10 specimens of *F*. *a*. *arctica* from Runde (Fig 3) showed that in almost all elements the mean greatest length for the Runde population was larger than the Dollsteinhola population. However, the comparison also showed that in many of the other measurements Dollsteinhola was on average slightly larger than Runde *F*. *a*. *arctica*. In essence, the Dollsteinhola population displayed shorter, yet sturdier skeletal elements than modern populations of *F*. *a*. *arctica*.

The Dollsteinhola assemblage spans ca. 3000 years from 6600 to 3600 BP [32] and covers both a warmer and colder period. Sea-surface temperatures in the Norwegian Sea were at maximum warmth around 9700–6700 BP, approximately 3–5°C warmer than present day [46]. After this period of maximum sea-surface temperature came a period of gradual cooling at a rate of 1°C every 1000 years until 3500 years BP [46]. Such shifts in climatic conditions likely affected Atlantic Puffin prey resources. Successful breeding seasons for Atlantic Puffin require a steady supply of small shoaling fish within a few tens of kilometres of the colony [22]. The prey of Atlantic Puffin is heavily dependent upon the availability of zooplankton, and with just slight changes to sea-surface temperatures these communities are heavily influenced [45,47,48]. The main prey of the more southern Norwegian Atlantic Puffin are the Lesser Sandeel (*Ammodytes marinus*), Atlantic Herring (*Clupea harengus*), Saithe (*Pollachius virens*) and Haddock (*Melanogrammus aeglefinus*) [14,49]. Warmer sea temperatures have been linked with negative impacts on Sandeel recruitment [50] and may have caused earlier blooms of phytoplankton, meaning important prey such as the Atlantic Herring spawn earlier. Through these indirect effects of temperature on Atlantic Puffin food resources, the fluctuating climate during the middle Holocene likely affected Atlantic Puffin breeding success, population size, and distributional range. Dollsteinhola is located close to the borders of the modern breeding ranges of both *F*. *a*. *arctica* and *F*. *a*. *grabae*. We consider it therefore likely that given the climatic oscillations and their concomitant effects on marine prey, breeding ranges of the two subspecies shifted north or south accordingly. The smaller specimens in Dollsteinhola are then likely to represent *F*. *a*. *grabae* specimens (or a smaller clinal population, if *F*. *a*. *grabae* is disregarded as a subspecies), while the larger ones would represent *F*. *a*. *arctica*. Puffins rarely come to land in the winter [51,52], but winter wreck events could result in the blending of both subspecies across the seasons. We have recorded juvenile Atlantic Puffin specimens from Dollsteinhola, suggesting that a breeding colony was present. If *F*. *a*. *grabae* was indeed breeding at Dollsteinhola, this would indicate a 145 Km shift north from the subspecies current breeding range (See Fig 1), as the closest modern population of *F*. *a*. *grabae* is at Utvær.

However, shifting ranges of *F*. *a*. *arctica* and *F*. *a*. *grabae* do not account for the differences in proportions that we observed in the Dollsteinhola material. The Dollsteinhola population displayed slightly shorter, sturdier skeletal elements than modern populations of *F*. *a*. *arctica*. These differences are most pronounced in the carpometacarpus. For wing propelled divers such as the Atlantic Puffin shorter wing bones (and potentially smaller flight feathers) reduce drag when diving and moving through the water [53–56]. This may have made the Dollsteinhola population better divers than their modern counterparts. Alternatively, shorter carpometacarpi may be linked to changes in flight; a number of studies have found the carpometacarpus to be particularly important as it is the attachment site for the primary flight feathers [57–59]. A shorter carpometacarpus might have led to a somewhat reduced flight efficiency. Whether this difference in proportions points towards functional difference in middle Holocene Atlantic Puffins or signal an influx of a smaller yet sturdier morphotype is unclear. Understanding the full scale of Atlantic Puffin morphometrics from a wider geographical and temporal scale would be helpful in exploring these specimens further.

## Conclusion

Our research has provided the first osteological study of modern *Fratercula arctica*. Our results show that the modern subspecies *F*. *a*. *naumanni* is generally recognisable osteologically from the two other subspecies by its larger size and different proportions. This supports the general view that *Fratercula arctica* is a polytypic species, whereby *F*. *a*. *naumanni* and *F*. *a*. *arctica* are subspecies. However, the subspecies *F*. *a*. *grabae* is less distinguishable from *F*. *a*. *arctica* and it might not represent a separate subspecies, but rather a north-south clinal variation. These results reflect ornithological observations. The archaeological specimens from Måsøy showed they were one population likely to be *F*. *a*. *arctica*, displaying no recognisable difference in size to modern populations, and suggesting that for this subspecies during the Little Ice Age there appeared to be no changes to the skeleton. The specimens from Dollsteinhola encompass the modern size range of both *F*. *a*. *arctica* and *F*. *a*. *grabae* but display slightly different proportions. It seems most likely that the Dollsteinhola assemblage represents a dynamic response to the climatic oscillations that occurred during the mid-Holocene whereby, *F*. *a*. *arctica* and *F*. *a*. *grabae* shifted north or south depending on the climatic conditions. This does not, however, explain the shorter and stockier proportions of the Dollsteinhola material. Understanding the full scale of Atlantic Puffin morphometrics from a wider geographical and temporal scale would be helpful in exploring these specimens further.

## Supporting information

S1 Table Dollsteinhola and Runde means values. Mean values of Dollsteinhola measurements and modern Runde *F*. *a*. *arctica* measurements.
(DOCX)

S1 Appendix List of modern specimens used for comparison. (DOCX)

S2 Appendix Modern subspecies ANOVA results. Details of the one-way ANOVA and Tukey’s pairwise results for the modern subspecies.
(DOCX)

S3 Appendix Modern subspecies and Archaeological specimens ANOVA results. Details of the one-way ANOVA and Tukey’s pairwise results for the modern subspecies in comparison to the archaeological specimens from Dollsteinhola and Måsøy.
(DOCX)

S1 Text Measurements. Description of all measurements featured within this paper.
(DOCX)
