# Supplementary material for: Size variation in mid-Holocene North Atlantic Puffins indicates a dynamic response to climate change
Source: PLoS One. 2021 Feb 24;16(2):e0246888. doi: 10.1371/journal.pone.0246888 (PMC7904199; doi:10.1371/journal.pone.0246888)
Supplement: S1 Table — Mean values of Dollsteinhola measurements and modern Runde F. a. arctica measurements. (DOCX) [file pone.0246888.s001.docx]

|  | **Runde (mean values)** | | | | | | | **Dollsteinhola (mean values)** | | | | | | |
| --- | --- | --- | --- | --- | --- | --- | --- | --- | --- | --- | --- | --- | --- | --- |
|  | Length | Proximal | | | Shaft | Distal | | Length | Proximal | | | Shaft | Distal | |
| Coracoid | **Lm** |  |  |  |  | **Bb** | **BF** | **Lm** |  |  |  |  | **Bb** | **BF** |
|  | 33.98 |  |  |  |  | 11.99 | 9.31* | 34.40 |  |  |  |  |  | 10.21* |
| Humerus | **GL** |  | **Bp** |  | **SC** | **Bd** | **KB** | **GL** | **Bp** |  |  | **SC** | **Bd** | **KB** |
|  | 63.71 |  | 14.69 |  | 3.04* | 6.62* | 3.88 | 63.38 | 14.54 |  |  | 3.27* | 6.92* | 3.95 |
| Ulna | **GL** | **Dip** | **Bp** | **Tp** | **SC** | **Did** |  | **GL** | **Dip** | **Bp** | **Tp** | **SC** | **Did** |  |
|  | 50.23 | 7.93 | 6.58 | 5.98 | 2.84 | 7.23 |  | 49.46 | 7.98 | 6.77 | 6.13 | 2.96 | 7.33 |  |
| Carpometacarpus | **GL** | **Bp** |  |  |  | **Did** | **HS** | **GL** | **Bp** |  |  |  | **Did** | **HS** |
|  | 33.94* | 7.76 |  |  |  | 4.92* | 3.76 | 32.84* | 8.00 |  |  |  | 5.30* | 3.67 |
| Femur | **GL** | **Bp** | **Dp** |  | **SC** | **Bd** | **Dd** | **GL** | **Bp** | **Dp** |  | **SC** | **Bd** | **Dd** |
|  | 38.40 | 7.63 | 4.98 |  | 2.86 | 6.86 | 5.74 | 37.46 | 7.63 | 4.92 |  | 2.98 | 7.05 | 5.95 |
| Tibiotarsus | **La** | **Dip** | **Bp** |  | **SC** | **Bd** | **Dd** | **La** | **Dip** | **Bp** |  | **SC** | **Bd** | **Dd** |
|  | 62.31 | 8.11 | 5.71 |  | 3.14 | 5.54* | 5.80 | 61.37 | 8.43 | 5.88 |  | 3.25 | 6.04* | 5.85 |
| Tarsometatarsus | **GL** | **Bp** |  |  | **SC** | **Bd** |  | **GL** | **Bp** |  |  | **SC** | **Bd** |  |
|  | 27.67 | 6.22 |  |  | 3.38 | 6.82 |  | 27.21 | 6.22 |  |  | 3.57 | 6.74 |  |

**S4 Table**. Dollsteinhola and Runde means values. Mean values of Dollsteinhola measurements and modern Runde *F. a. arctica* measurements.

Comparison of mean values for modern *F. a. arctica* from Runde and the *F. arctica* specimens from Dollsteinhola. Highlighted values (light grey) represent the higher mean, * are the significantly different means.
