# Supplementary material for: Size variation in mid-Holocene North Atlantic Puffins indicates a dynamic response to climate change
Source: PLoS One. 2021 Feb 24;16(2):e0246888. doi: 10.1371/journal.pone.0246888 (PMC7904199; doi:10.1371/journal.pone.0246888)
Supplement: S1 Appendix — (DOCX) [file pone.0246888.s002.docx]

# **Appendix**

**Appendix 1. List of modern specimens used for comparison.** *Fratercula arctica arctica*; B 462, B 465, B 466, B 467, B 468, B 469, B 470, B 471, B 1851, B 1852, B 2763, BM 5210, BM 7114, BM 7115, BM 10577, BM 10578, NHMD 223202, NHMD 223209, NHMD 223210, NHMD 223215, NHMD 223216, NHMD 223217, NHMD 223219, NHMD 223220, NHMD 223222, NHMD 223223, NHMD 223224, NHMD 223225, NHMD 223226, NHMD 223227, NHMD 223228, NHMD 223229, NHMD 223230, NHMD 223231, NHMD 223232, NHMD 223233, NHMD 223236, NHMD 223237, NHMD 223238, NHMD 223239, NHMD 223244, NHMD 223245, NHMD 223253, NHMD 223256, NHMD 223257, NHMD 223260, NHMD 223262, NHMD 223264.

*Fratercula arctica grabae* from diagnostic external characteristics during preparation; CN 248, NHMD 223207, NHMUK S/1973.66.92, MHNG 856.011, MHNG 856.012. *Fratercula arctica grabae* from location during breeding season; B 3052, NHMD 223250, NHMD 223251, NHMD 223252, NHMD 223258, NHMD 223263 (All recorded as *F. a. arctica* on the museum labels. However, their location in *F. a. grabae* breeding colonies during the breeding season is why we have reclassified them for this paper).

*Fratercula arctica naumanni* from diagnostic external characteristics during preparation; B 981, B 982, B 1829, BM 10334, BM 10335, BM 10336, BM 10339, BM 10342, BM 10343, BM 10344, BM 10345, BM 10346, BM 10347, BM 10348, NHMUK 1931.8.2.17. *Fratercula arctica naumanni* from location during breeding season; BM 10341 (recorded as *F. a. arctica* on the museum label. However, its location in *F. a. naumanni* breeding colonies during the breeding season is why we have reclassified it for this paper).

B and BM numbers relate to the University Museum of Bergen. NHMD and CN numbers relate to the Natural History Museum of Denmark. MHNG numbers are from the Natural History Museum of Geneva. NHMUK numbers are related to the Natural History Museum at Tring.
