# Supplementary material for: Size variation in mid-Holocene North Atlantic Puffins indicates a dynamic response to climate change
Source: PLoS One. 2021 Feb 24;16(2):e0246888. doi: 10.1371/journal.pone.0246888 (PMC7904199; doi:10.1371/journal.pone.0246888)
Supplement: S2 Appendix — Details of the one-way ANOVA and Tukey’s pairwise results for the modern subspecies. (DOCX) [file pone.0246888.s003.docx]

**S2 Appendix**. Modern subspecies ANOVA results. Details of the one-way ANOVA and Tukey’s pairwise results for the modern subspecies.

# Coracoid

Coracoid Lm shows the groups are normally distributed (Shapiro-Wilk p = 0.497). The Levene’s test for homogeneity of variance reports a p value of 0.02, this means that the variances are unequal, this compromises the assumptions of a one-way ANOVA. As a result Welch’s F test (in the case of unequal variances) was consulted and included in Table 1 along with the one-way ANOVA results, as the p value of the Welch’s test was significant (p=<.001) it was possible to continue to Tukey’s pairwise (Table 4). The coracoid Bb groups are also normally distributed (Shapiro-Wilk p = 0.9818). Levene’s test for homogeneity reports a p value of 0.09 for the equality of variances, so there is no significant difference. This means the assumptions of the ANOVA are safely met. Table 2 presents the results of the one-way ANOVA in standard form and Table 5 shows the Tukey’s pairwise results. The coracoid BF measurement shows the groups are normally distributed (Shapiro-wilk p = 0.198). Levene’s test for homogeneity reports a p value of 0.547 for the equality of variances, so there is no significant difference. The sample sizes do differ. However, the assumptions of the ANOVA are safely met. Table 3 presents the results of the one-way ANOVA in standard form and Table 6 shows the Tukey’s pairwise results.

| **Table 1**, Coracoid Lm one-way ANOVA | | | | | |
| --- | --- | --- | --- | --- | --- |
|  | **Sum of sqrs** | **df** | **Mean square** | ***F*** | ***p*(same)** |
| **Between groups** | 126.461 | 2 | 63.2305 | 34.72 | 3.344E-11 |
| **Within groups** | 127.471 | 70 | 1.82102 |  |  |
| **Welch *F* test** |  | 21.47 |  | 73.08 | 2.624E-10 |

One-way ANOVA results for the medial length (Lm) of the coracoid. Taking into account only modern material.

| **Table 2**, Coracoid Bb one-way ANOVA | | | | | |
| --- | --- | --- | --- | --- | --- |
|  | **Sum of sqrs** | **df** | **Mean square** | ***F*** | ***p*(same)** |
| **Between groups** | 16.1684 | 2 | 8.08421 | 10.98 | 7.022E-05 |
| **Within groups** | 52.2935 | 71 | 0.736528 |  |  |

One-way ANOVA results for the basal breadth (Bb) of the coracoid. Taking into account only modern material.

| **Table 3**, Coracoid BF one-way ANOVA | | | | | |
| --- | --- | --- | --- | --- | --- |
|  | **Sum of sqrs** | **df** | **Mean square** | ***F*** | ***p*(same)** |
| **Between groups** | 15.2398 | 2 | 7.61989 | 18.35 | 3.76E-07 |
| **Within groups** | 29.4782 | 71 | 0.415186 |  |  |

One-way ANOVA results for the basal articular surface (BF) of the coracoid. Taking into account only modern material.

| **Table 4**, Coracoid Lm ANOVA – Tukey’s pairwise | | | |  | **Table 5**, Coracoid Bb ANOVA – Tukey’s pairwise | | | |
| --- | --- | --- | --- | --- | --- | --- | --- | --- |
|  | ***F. a. grabae*** | ***F. a. arctica*** | ***F. a. naumanni*** |  |  | ***F. a. grabae*** | ***F. a. arctica*** | ***F. a. naumanni*** |
| ***F. a. grabae*** | - | 0.185 | <.001 |  | ***F. a. grabae*** | - | 0.721 | <.001 |
| ***F. a. arctica*** | 2.51 | - | <.001 |  | ***F. a. arctica*** | 1.092 | - | <.001 |
| ***F. a. naumanni*** | 9.94 | 10.77 | - |  | ***F. a. naumanni*** | 5.358 | 6.166 | - |
|  |  |  |  |  |  |  |  |  |
|  |  |  |  |  |  |  |  |  |
| **Table 6**, Coracoid BF ANOVA – Tukey’s pairwise | | | |  |  | | | |
|  | ***F. a. grabae*** | ***F. a. arctica*** | ***F. a. naumanni*** |  |  |  |  |  |
| ***F. a. grabae*** | - | 0.071 | <.001 |  |  |  |  |  |
| ***F. a. arctica*** | 3.171 | - | <.001 |  |  |  |  |  |
| ***F. a. naumanni*** | 7.839 | 7.128 | - |  |  |  |  |  |

# Humerus

The humerus GL shows the groups are normally distributed (Shapiro-wilk p 0.172). The Levene’s test for homogeneity of variance reports a p value of 0.05, this means that the variances are unequal, this compromises the assumptions of a one-way ANOVA. As a result Welch’s F test (in the case of unequal variances) was consulted and included in Table 7 along with the one-way ANOVA results, as the p value of the Welch’s test was significant (p=<.001) it was possible to continue to Tukey’s pairwise (Table 12). The Humerus Bp is normally distributed (Shapiro-Wilk p value of 0.853). Levene’s test for homogeneity reports a p value of 0.330 for the equality of variances, so there is no significant difference. This means the assumptions of the ANOVA are safely met. Table 8 presents the results of the one-way ANOVA in standard form and Table 13 shows the Tukey’s pairwise results. The humerus SC normal distribution was distorted by an outlier (a single large SC measure on *F. a. grabae* specimen) giving a Shapiro-wilk p value of 0.002. Despite this the graphs show that the rest of the data has a normal distribution. The Levene’s test for homogeneity reports a p value of 0.415 for the equality of variances, so there is no significant difference. Despite the outlier all other assumptions of the ANOVA are met, and we therefore continued to run the ANOVA, the results of which are presented in Table 9 and Tukey’s pairwise results in Table 14. The Humerus Bd is normally distributed (Shapiro-Wilk p value of 0.926). Levene’s test for homogeneity reports a p value of 0.07 for the equality of variances, so there is no significant difference. This means all the assumptions of the ANOVA are safely met. Table 10 presents the results of the one-way ANOVA in standard form and Table 15 shows the Tukey’s pairwise results. The Humerus KB is normally distributed (Shapiro-Wilk p value of 0.272). Levene’s test for homogeneity reports a p value of <.001, this means that the variances are unequal, this compromises the assumptions of a one-way ANOVA. As a result Welch’s F test (in the case of unequal variances) was consulted and included in Table 11 along with the one-way ANOVA results, as the p value of the Welch’s test was significant (p=<.001) it was possible to continue to Tukey’s pairwise (Table 16).

| **Table 7**, Humerus GL one-way ANOVA | | | | | |
| --- | --- | --- | --- | --- | --- |
|  | **Sum of sqrs** | **df** | **Mean square** | ***F*** | ***p*(same)** |
| **Between groups** | 327.192 | 2 | 163.596 | 42.71 | 1.099E-12 |
| **Within groups** | 256.618 | 67 | 3.83011 |  |  |
| **Welch *F* test** |  | 19.38 |  | 41.79 | 9.389E-08 |

One-way ANOVA results for the greatest length (GL) of the humerus. Taking into account only modern material.

| **Table 8**, Humerus Bp one-way ANOVA | | | | | |
| --- | --- | --- | --- | --- | --- |
|  | **Sum of sqrs** | **df** | **Mean square** | ***F*** | ***p*(same)** |
| **Between groups** | 22.1475 | 2 | 11.0737 | 25.49 | 4.259E-09 |
| **Within groups** | 31.2762 | 72 | 0.434392 |  |  |

One-way ANOVA results for the breadth of the proximal end (Bp) of the humerus. Taking into account only modern material.

| **Table 9**, Humerus SC one-way ANOVA | | | | | |
| --- | --- | --- | --- | --- | --- |
|  | **Sum of sqrs** | **df** | **Mean square** | ***F*** | ***p*(same)** |
| **Between groups** | 0.921719 | 2 | 0.460859 | 12.38 | 2.5E-05 |
| **Within groups** | 2.60669 | 70 | 0.0372384 |  |  |

One-way ANOVA results for the smallest breadth of the corpus (SC) of the humerus. Taking into account only modern material.

| **Table 10**, Humerus Bd one-way ANOVA | | | | | |
| --- | --- | --- | --- | --- | --- |
|  | **Sum of sqrs** | **df** | **Mean square** | ***F*** | ***p*(same)** |
| **Between groups** | 2.88361 | 2 | 1.4418 | 16.2 | 1.769E-06 |
| **Within groups** | 6.05387 | 68 | 0.0890275 |  |  |

One-way ANOVA results for the breadth of the distal end (Bd) of the humerus. Taking into account only modern material.

| **Table 11**, Humerus KB one-way ANOVA | | | | | |
| --- | --- | --- | --- | --- | --- |
|  | **Sum of sqrs** | **df** | **Mean square** | ***F*** | ***p*(same)** |
| **Between groups** | 1.5607 | 2 | 0.780352 | 13.26 | 1.369E-05 |
| **Within groups** | 4.00086 | 68 | 0.0588362 |  |  |
| **Welch *F* test** |  | 20.08 |  | 14.31 | 0.0001372 |

One-way ANOVA results for the smallest depth of the distal shaft (KB) of the humerus. Taking into account only modern material.

| **Table 12**, Humerus GL ANOVA - Tukey’s pairwise | | | |  | **Table 13**, Humerus Bp ANOVA - Tukey’s pairwise | | | |
| --- | --- | --- | --- | --- | --- | --- | --- | --- |
|  | ***F. a. grabae*** | ***F. a. arctica*** | ***F. a. naumanni*** |  |  | ***F. a. grabae*** | ***F. a. arctica*** | ***F. a. naumanni*** |
| ***F. a. grabae*** | - | <.001 | <.001 |  | ***F. a. grabae*** | - | <.001 | <.001 |
| ***F. a. arctica*** | 5.292 | - | <.001 |  | ***F. a. arctica*** | 5.672 | - | <.001 |
| ***F. a. naumanni*** | 12.41 | 10.53 | - |  | ***F. a. naumanni*** | 9.951 | 6.933 | - |
|  |  |  |  |  |  |  |  |  |
|  |  |  |  |  |  |  |  |  |
| **Table 14**, Humerus SC ANOVA - Tukey’s pairwise | | | |  | **Table 15**, Humerus Bd ANOVA - Tukey’s pairwise | | | |
|  | ***F. a. grabae*** | ***F. a. arctica*** | ***F. a. naumanni*** |  |  | ***F. a. grabae*** | ***F. a. arctica*** | ***F. a. naumanni*** |
| ***F. a. grabae*** | - | 0.554 | <.001 |  | ***F. a. grabae*** | - | 0.133 | <.001 |
| ***F. a. arctica*** | 1.472 | - | <.001 |  | ***F. a. arctica*** | 2.755 | - | <.001 |
| ***F. a. naumanni*** | 5.987 | 6.378 | - |  | ***F. a. naumanni*** | 7.381 | 6.722 | - |
|  |  |  |  |  |  |  |  |  |
|  |  |  |  |  |  |  |  |  |
| **Table 16**, Humerus KB ANOVA - Tukey’s pairwise | | | |  |  | | | |
|  | ***F. a. grabae*** | ***F. a. arctica*** | ***F. a. naumanni*** |  |  |  |  |  |
| ***F. a. grabae*** | - | 0.292 | <.001 |  |  |  |  |  |
| ***F. a. arctica*** | 2.138 | - | <.001 |  |  |  |  |  |
| ***F. a. naumanni*** | 6.522 | 6.281 | - |  |  |  |  |  |

# Ulna

The ulna GL shows the groups are normally distributed (Shapiro-wilk p 0.759). The Levene’s test for homogeneity of variance reports a p value of 0.008, this means that the variances are unequal, this compromises the assumptions of a one-way ANOVA. As a result Welch’s F test (in the case of unequal variances) was consulted and included in Table 17 along with the one-way ANOVA results, as the p value of the Welch’s test was significant (p=<.001) it was possible to continue to Tukey’s pairwise (Table 23). The ulna Dip is normally distributed (Shapiro-Wilk p value of 0.991). Levene’s test for homogeneity reports a p value of 0.168 for the equality of variances, so there is no significant difference. This means all the assumptions of the ANOVA are safely met. Table 18 presents the results of the one-way ANOVA in standard form and Table 24 shows the Tukey’s pairwise results. The ulna Bp is normally distributed (Shapiro-Wilk p value of 0.220). Levene’s test for homogeneity reports a p value of 0.266 for the equality of variances, so there is no significant difference. This means all the assumptions of the ANOVA are safely met. Table 19 presents the results of the one-way ANOVA in standard form and Table 25 shows the Tukey’s pairwise results. The ulna Tp measurement shows a relatively normal distribution when glancing at the residual graphs, however, Shapiro-wilks test for normality gives a p value of 0.05. As the graphs show the data has a fairly normal distribution we continue to use the one-way ANOVA and do not move to a non-parametric test. The Levene’s test for homogeneity reports a p value of <.001 for the equality of variances, this means that the variances are unequal, this compromises the assumptions of a one-way ANOVA. As a result Welch’s F test (in the case of unequal variances) was consulted and included in Table 20 along with the one-way ANOVA results, as the p value of the Welch’s test was significant (p=<.001) it was possible to continue to Tukey’s pairwise (Table 26). The ulna SC measurement shows a relatively normal distribution when glancing at the residual graphs, however, two large measurements for the subspecies *F. a. grabae* have led to a Shapiro-wilks p value of 0.003. As the graphs show the data has a fairly normal distribution we continue to use the one-way ANOVA and do not move to a non-parametric test. The Levene’s test for homogeneity reports a p value of <.001 for the equality of variances, this means that the variances are unequal, this compromises the assumptions of a one-way ANOVA. As a result, Welch’s F test (in the case of unequal variances) was consulted and included in Table 21 along with the one-way ANOVA results, as the p value of the Welch’s test was significant (p=<.001) it was possible to continue to Tukey’s pairwise (Table 27). The ulna Did is normally distributed (Shapiro-Wilk p value of 0.986). Levene’s test for homogeneity reports a p value of 0.04 for the equality of variances, this means that the variances are unequal, this compromises the assumptions of a one-way ANOVA. As a result, Welch’s F test (in the case of unequal variances) was consulted and included in Table 22 along with the one-way ANOVA results, as the p value of the Welch’s test was significant (p=<.001) it was possible to continue to Tukey’s pairwise (Table 28).

| **Table 17**, Ulna GL one-way ANOVA | | | | | |
| --- | --- | --- | --- | --- | --- |
|  | **Sum of sqrs** | **df** | **Mean square** | ***F*** | ***p*(same)** |
| **Between groups** | 167.146 | 2 | 83.573 | 40.81 | 1.159E-10 |
| **Within groups** | 88.0552 | 43 | 2.0478 |  |  |
| **Welch *F* test** |  | 11.44 |  | 50.44 | 2.112E-06 |

One-way ANOVA results for the greatest length (GL) of the ulna. Taking into account only modern material.

| **Table 18**, Ulna Dip one-way ANOVA | | | | | |
| --- | --- | --- | --- | --- | --- |
|  | **Sum of sqrs** | **df** | **Mean square** | ***F*** | ***p*(same)** |
| **Between groups** | 4.59556 | 2 | 2.29778 | 10.67 | 0.0001786 |
| **Within groups** | 9.04174 | 42 | 0.215279 |  |  |

One-way ANOVA results for the diagonal of the proximal end (Dip) of the ulna. Taking into account only modern material.

| **Table 19**, Ulna Bp one-way ANOVA | | | | | |
| --- | --- | --- | --- | --- | --- |
|  | **Sum of sqrs** | **df** | **Mean square** | ***F*** | ***p*(same)** |
| **Between groups** | 3.27703 | 2 | 1.63851 | 19.13 | 1.243E-06 |
| **Within groups** | 3.59809 | 42 | 0.0856688 |  |  |

One-way ANOVA results for the breadth of the proximal end (Bp) of the ulna. Taking into account only modern material.

| **Table 20**, Ulna Tp one-way ANOVA | | | | | |
| --- | --- | --- | --- | --- | --- |
|  | **Sum of sqrs** | **df** | **Mean square** | ***F*** | ***p*(same)** |
| **Between groups** | 3.34374 | 2 | 1.67187 | 14.2 | 1.943E-05 |
| **Within groups** | 4.94386 | 42 | 0.117711 |  |  |
| **Welch *F* test** |  | 11.5 |  | 22.01 | 0.0001172 |

One-way ANOVA results for the depth of the proximal end (Tp) of the ulna. Taking into account only modern material.

| **Table 21**, Ulna SC one-way ANOVA | | | | | |
| --- | --- | --- | --- | --- | --- |
|  | **Sum of sqrs** | **df** | **Mean square** | ***F*** | ***p*(same)** |
| **Between groups** | 1.04119 | 2 | 0.520596 | 8.69 | 0.0006287 |
| **Within groups** | 2.75565 | 46 | 0.0599054 |  |  |
| **Welch *F* test** |  | 11.26 |  | 15.38 | 0.0006029 |

One-way ANOVA results for the smallest breadth of the corpus (SC) of the ulna. Taking into account only modern material.

| **Table 22**, Ulna Did one-way ANOVA | | | | | |
| --- | --- | --- | --- | --- | --- |
|  | **Sum of sqrs** | **df** | **Mean square** | ***F*** | ***p*(same)** |
| **Between groups** | 4.32572 | 2 | 2.16286 | 29.54 | 5.614E-09 |
| **Within groups** | 3.36853 | 46 | 0.0732289 |  |  |
| **Welch *F* test** |  | 13.89 |  | 41.84 | 1.321E-06 |

One-way ANOVA results for the diagonal of the distal end (Did) of the ulna. Taking into account only modern material.

| **Table 23**, Ulna GL ANOVA - Tukey’s pairwise | | | |  | **Table 24**, Ulna Dip ANOVA - Tukey’s pairwise | | | |
| --- | --- | --- | --- | --- | --- | --- | --- | --- |
|  | ***F. a. grabae*** | ***F. a. arctica*** | ***F. a. naumanni*** |  |  | ***F. a. grabae*** | ***F. a. arctica*** | ***F. a. naumanni*** |
| ***F. a. grabae*** | - | 0.969 | <.001 |  | ***F. a. grabae*** | - | 0.014 | 0.970 |
| ***F. a. arctica*** | 0.338 | - | <.001 |  | ***F. a. arctica*** | 4.156 | - | <.001 |
| ***F. a. naumanni*** | 8.221 | 12.47 | - |  | ***F. a. naumanni*** | 0.330 | 5.882 | - |
|  |  |  |  |  |  |  |  |  |
|  |  |  |  |  |  |  |  |  |
| **Table 25**, Ulna Bp ANOVA - Tukey’s pairwise | | | |  | **Table 26**, Ulna Tp ANOVA - Tukey’s pairwise | | | |
|  | ***F. a. grabae*** | ***F. a. arctica*** | ***F. a. naumanni*** |  |  | ***F. a. grabae*** | ***F. a. arctica*** | ***F. a. naumanni*** |
| ***F. a. grabae*** | - | 0.128 | <.001 |  | ***F. a. grabae*** | - | <.001 | 0.904 |
| ***F. a. arctica*** | 2.807 | - | <.001 |  | ***F. a. arctica*** | 5.512 | - | <.001 |
| ***F. a. naumanni*** | 7.658 | 7.385 | - |  | ***F. a. naumanni*** | 0.605 | 6.299 | - |
|  |  |  |  |  |  |  |  |  |
|  |  |  |  |  |  |  |  |  |
| **Table 27**, Ulna SC ANOVA - Tukey’s pairwise | | | |  | **Table 28**, Ulna Did ANOVA - Tukey’s pairwise | | | |
|  | ***F. a. grabae*** | ***F. a. arctica*** | ***F. a. naumanni*** |  |  | ***F. a. grabae*** | ***F. a. arctica*** | ***F. a. naumanni*** |
| ***F. a. grabae*** | - | 0.026 | 1 |  | ***F. a. grabae*** | - | 0.991 | <.001 |
| ***F. a. arctica*** | 3.779 | - | <.001 |  | ***F. a. arctica*** | 0.182 | - | <.001 |
| ***F. a. naumanni*** | 0.035 | 5.353 | - |  | ***F. a. naumanni*** | 6.775 | 10.54 | - |

# Carpometacarpus

The carpometacarpus GL shows the groups are normally distributed (Shapiro-wilk p 0.844). The Levene’s test for homogeneity of variance reports a p value of 0.04, this means that the variances are unequal, this compromises the assumptions of a one-way ANOVA. As a result, Welch’s F test (in the case of unequal variances) was consulted and included in Table 29 along with the one-way ANOVA results, as the p value of the Welch’s test was significant (p=<.001) it was possible to continue to Tukey’s pairwise (Table 33). The carpometacarpus Bp is normally distributed (Shapiro-Wilk p value of 0.681). Levene’s test for homogeneity reports a p value of 0.592 for the equality of variances, so there is no significant difference. This means all the assumptions of the ANOVA are safely met. Table 30 presents the results of the one-way ANOVA in standard form and Table 34 shows the Tukey’s pairwise results. The carpometacarpus Did is normally distributed (Shapiro-Wilk p value of 0.973). Levene’s test for homogeneity reports a p value of 0.194 for the equality of variances, so there is no significant difference. This means all the assumptions of the ANOVA are safely met. Table 31 presents the results of the one-way ANOVA in standard form and Table 35 shows the Tukey’s pairwise results. The carpometacarpus HS is normally distributed (Shapiro-wilk p value of 0.712). The Levene’s test for homogeneity of variance reports a p value of <.001, this means that the variances are unequal, this compromises the assumptions of a one-way ANOVA. As a result, Welch’s F test (in the case of unequal variances) was consulted and included in Table 32 along with the one-way ANOVA results, as the p value of the Welch’s test was significant (0.009) it was possible to continue to Tukey’s pairwise (Table 36).

| **Table 29**, Carpometacarpus GL one-way ANOVA | | | | | |
| --- | --- | --- | --- | --- | --- |
|  | **Sum of sqrs** | **df** | **Mean square** | ***F*** | ***p*(same)** |
| **Between groups** | 94.0161 | 2 | 47.008 | 49.39 | 3.53E-12 |
| **Within groups** | 43.7856 | 46 | 0.951861 |  |  |
| **Welch *F* test** |  | 12.35 |  | 60.31 | 4.239E-07 |

One-way ANOVA results for the greatest length (GL) of the carpometacarpus. Taking into account only modern material.

| **Table 30**, Carpometacarpus Bp one-way ANOVA | | | | | |
| --- | --- | --- | --- | --- | --- |
|  | **Sum of sqrs** | **df** | **Mean square** | ***F*** | ***p*(same)** |
| **Between groups** | 3.89637 | 2 | 1.94818 | 12.54 | 4.5E-05 |
| **Within groups** | 7.14644 | 46 | 0.155357 |  |  |

One-way ANOVA results for the breadth of the proximal end (Bp) of the carpometacarpus. Taking into account only modern material.

| **Table 31**, Carpometacarpus Did one-way ANOVA | | | | | |
| --- | --- | --- | --- | --- | --- |
|  | **Sum of sqrs** | **df** | **Mean square** | ***F*** | ***p*(same)** |
| **Between groups** | 2.78986 | 2 | 1.39493 | 16.01 | 5.275E-06 |
| **Within groups** | 4.00744 | 46 | 0.0871182 |  |  |

One-way ANOVA results for the diagonal of the distal end (Did) of the carpometacarpus. Taking into account only modern material.

| **Table 32**, Carpometacarpus HS one-way ANOVA | | | | | |
| --- | --- | --- | --- | --- | --- |
|  | **Sum of sqrs** | **df** | **Mean square** | ***F*** | ***p*(same)** |
| **Between groups** | 0.899687 | 2 | 0.449844 | 5.269 | 0.008704 |
| **Within groups** | 3.9275 | 46 | 0.0853804 |  |  |
| **Welch *F* test** |  | 11.85 |  | 7.105 | 0.009374 |

One-way ANOVA results for the height of the symphysis (HS) of the carpometacarpus. Taking into account only modern material.

| **Table 33**, Carpometacarpus GL ANOVA - Tukey’s pairwise | | | |  | **Table 34**, Carpometacarpus Bp ANOVA - Tukey’s pairwise | | | |
| --- | --- | --- | --- | --- | --- | --- | --- | --- |
|  | ***F. a. grabae*** | ***F. a. arctica*** | ***F. a. naumanni*** |  |  | ***F. a. grabae*** | ***F. a. arctica*** | ***F. a. naumanni*** |
| ***F. a. grabae*** | - | 0.963 | <.001 |  | ***F. a. grabae*** | - | 0.258 | 0.184 |
| ***F. a. arctica*** | 0.370 | - | <.001 |  | ***F. a. arctica*** | 2.257 | - | <.001 |
| ***F. a. naumanni*** | 8.656 | 13.66 | - |  | ***F. a. naumanni*** | 2.531 | 7.069 | - |
|  |  |  |  |  |  |  |  |  |
|  |  |  |  |  |  |  |  |  |
| **Table 35**, Carpometacarpus Did ANOVA - Tukey’s pairwise | | | |  | **Table 36**, Carpometacarpus HS ANOVA - Tukey’s pairwise | | | |
|  | ***F. a. grabae*** | ***F. a. arctica*** | ***F. a. naumanni*** |  |  | ***F. a. grabae*** | ***F. a. arctica*** | ***F. a. naumanni*** |
| ***F. a. grabae*** | - | 0.961 | <.001 |  | ***F. a. grabae*** | - | 0.218 | 0.893 |
| ***F. a. arctica*** | 0.379 | - | <.001 |  | ***F. a. arctica*** | 2.399 | - | 0.009 |
| ***F. a. naumanni*** | 5.38 | 7.62 | - |  | ***F. a. naumanni*** | 0.640 | 4.403 | - |

# Femur

The femur GL shows the groups are normally distributed (Shapiro-wilk p 0.160). The Levene’s test for homogeneity of variance reports a p value of 0.08 for the equality of variances, so there is no significant difference. This means all the assumptions of the ANOVA are safely met. Table 37 presents the results of the one-way ANOVA in standard form and Table 43 shows the Tukey’s pairwise results. The femur Bp shows the groups are normally distributed (Shapiro-wilk p 0.315). The Levene’s test for homogeneity of variance reports a p value of 0.629 for the equality of variances, so there is no significant difference. This means all the assumptions of the ANOVA are safely met. Table 38 presents the results of the one-way ANOVA in standard form and Table 44 shows the Tukey’s pairwise results. The femur Dp shows the groups are normally distributed (Shapiro-wilk p value of 0.330). The Levene’s test for homogeneity of variance reports a p value of 0.508 for the equality of variances, so there is no significant difference. This means all the assumptions of the ANOVA are safely met. Table 39 presents the results of the one-way ANOVA in standard form and Table 45 shows the Tukey’s pairwise results. The femur SC shows the groups are normally distributed (Shapiro-wilk p value of 0.321). The Levene’s test for homogeneity of variance reports a p value of 0.507 for the equality of variances, so there is no significant difference. This means all the assumptions of the ANOVA are safely met. Table 40 presents the results of the one-way ANOVA in standard form and Table 46 shows the Tukey’s pairwise results. The femur Bd shows the groups are normally distributed (Shapiro-wilk p value of 0.799). The Levene’s test for homogeneity of variance reports a p value of 0.578 for the equality of variances, so there is no significant difference. This means all the assumptions of the ANOVA are safely met. Table 41 presents the results of the one-way ANOVA in standard form and Table 47 shows the Tukey’s pairwise results. The femur Dd is normally distributed (Shapiro-Wilk p value of 0.649). Levene’s test for homogeneity reports a p value of 0.005 for the equality of variances, this means that the variances are unequal, this compromises the assumptions of a one-way ANOVA. As a result, Welch’s F test (in the case of unequal variances) was consulted and included in Table 42 along with the one-way ANOVA results, as the p value of the Welch’s test was significant (p=<.001) it was possible to continue to Tukey’s pairwise (Table 48).

| **Table 37**, Femur GL one-way ANOVA | | | | | |
| --- | --- | --- | --- | --- | --- |
|  | **Sum of sqrs** | **df** | **Mean square** | ***F*** | ***p*(same)** |
| **Between groups** | 91.3539 | 2 | 45.677 | 18.11 | 4.966E-07 |
| **Within groups** | 171.545 | 68 | 2.52273 |  |  |

One-way ANOVA results for the greatest length (GL) of the femur. Taking into account only modern material.

| **Table 38**, Femur Bp one-way ANOVA | | | | | |
| --- | --- | --- | --- | --- | --- |
|  | **Sum of sqrs** | **df** | **Mean square** | ***F*** | ***p*(same)** |
| **Between groups** | 6.1971 | 2 | 3.09855 | 18.3 | 4.385E-07 |
| **Within groups** | 11.5153 | 68 | 0.169343 |  |  |

One-way ANOVA results for the breadth of the proximal end (Bp) of the femur. Taking into account only modern material.

| **Table 39,** Femur Dp one-way ANOVA | | | | | |
| --- | --- | --- | --- | --- | --- |
|  | **Sum of sqrs** | **df** | **Mean square** | ***F*** | ***p*(same)** |
| **Between groups** | 2.04558 | 2 | 1.02279 | 12.21 | 2.952E-05 |
| **Within groups** | 5.69728 | 68 | 0.0837835 |  |  |

One-way ANOVA results for the depth of the proximal end (Dp) of the femur. Taking into account only modern material.

| **Table 40**, Femur SC one-way ANOVA | | | | | |
| --- | --- | --- | --- | --- | --- |
|  | **Sum of sqrs** | **df** | **Mean square** | ***F*** | ***p*(same)** |
| **Between groups** | 0.552391 | 2 | 0.276196 | 9.367 | 0.0002588 |
| **Within groups** | 1.97564 | 67 | 0.0294872 |  |  |

One-way ANOVA results for the smallest breadth of the corpus (SC) of the femur. Taking into account only modern material.

| **Table 41**, Femur Bd one-way ANOVA | | | | | |
| --- | --- | --- | --- | --- | --- |
|  | **Sum of sqrs** | **df** | **Mean square** | ***F*** | ***p*(same)** |
| **Between groups** | 3.25207 | 2 | 1.62604 | 11.72 | 4.232E-05 |
| **Within groups** | 9.43412 | 68 | 0.138737 |  |  |

One-way ANOVA results for the breadth of the distal end (Bd) of the femur. Taking into account only modern material.

| **Table 42**, Femur Dd one-way ANOVA | | | | | |
| --- | --- | --- | --- | --- | --- |
|  | **Sum of sqrs** | **df** | **Mean square** | ***F*** | ***p*(same)** |
| **Between groups** | 5.05622 | 2 | 2.52811 | 17.11 | 9.907E-07 |
| **Within groups** | 9.89736 | 67 | 0.147722 |  |  |
| **Welch *F* test** |  | 16.93 |  | 19.29 | 4.307E-05 |

One-way ANOVA results for the depth of the distal end (Dd) of the femur. Taking into account only modern material.

| **Table 43**, Femur GL ANOVA - Tukey’s pairwise | | | |  | **Table 44**, Femur Bp ANOVA - Tukey’s pairwise | | | |
| --- | --- | --- | --- | --- | --- | --- | --- | --- |
|  | ***F. a. grabae*** | ***F. a. arctica*** | ***F. a. naumanni*** |  |  | ***F. a. grabae*** | ***F. a. arctica*** | ***F. a. naumanni*** |
| ***F. a. grabae*** | - | 0.064 | <.001 |  | ***F. a. grabae*** | - | 0.002 | <.001 |
| ***F. a. arctica*** | 3.239 | - | <.001 |  | ***F. a. arctica*** | 5.02 | - | <.001 |
| ***F. a. naumanni*** | 7.607 | 7.101 | - |  | ***F. a. naumanni*** | 8.333 | 5.833 | - |
|  |  |  |  |  |  |  |  |  |
|  |  |  |  |  |  |  |  |  |
| **Table 45**, Femur Dp ANOVA - Tukey’s pairwise | | | |  | **Table 46**, Femur SC ANOVA - Tukey’s pairwise | | | |
|  | ***F. a. grabae*** | ***F. a. arctica*** | ***F. a. naumanni*** |  |  | ***F. a. grabae*** | ***F. a. arctica*** | ***F. a. naumanni*** |
| ***F. a. grabae*** | - | 0.832 | <.001 |  | ***F. a. grabae*** | - | 0.329 | <.001 |
| ***F. a. arctica*** | 0.817 | - | <.001 |  | ***F. a. arctica*** | 2.03 | - | <.001 |
| ***F. a. naumanni*** | 5.169 | 6.653 | - |  | ***F. a. naumanni*** | 5.325 | 5.266 | - |
|  |  |  |  |  |  |  |  |  |
|  |  |  |  |  |  |  |  |  |
| **Table 47**, Femur Bd ANOVA - Tukey’s pairwise | | | |  | **Table 48**, Femur Dd ANOVA - Tukey’s pairwise | | | |
|  | ***F. a. grabae*** | ***F. a. arctica*** | ***F. a. naumanni*** |  |  | ***F. a. grabae*** | ***F. a. arctica*** | ***F. a. naumanni*** |
| ***F. a. grabae*** | - | 0.073 | <.001 |  | ***F. a. grabae*** | - | 0.002 | <.001 |
| ***F. a. arctica*** | 3.156 | - | <.001 |  | ***F. a. arctica*** | 5.034 | - | <.001 |
| ***F. a. naumanni*** | 6.368 | 5.356 | ­- |  | ***F. a. naumanni*** | 8.11 | 5.455 | - |

# Tibiotarsus

The tibiotarsus La shows the groups are normally distributed (Shapiro-wilk p 0.876). The Levene’s test for homogeneity of variance reports a p value of 0.002 for the equality of variances, this means that the variances are unequal, this compromises the assumptions of a one-way ANOVA. As a result, Welch’s F test (in the case of unequal variances) was consulted and included in Table 49 along with the one-way ANOVA results, as the p value of the Welch’s test was significant (p=<.001) it was possible to continue to Tukey’s pairwise (Table 55). The tibiotarsus Dip shows the groups are normally distributed (Shapiro-wilk p 0.665). The Levene’s test for homogeneity of variance reports a p value of 0.188 for the equality of variances, so there is no significant difference. This means all the assumptions of the ANOVA are safely met. Table 50 presents the results of the one-way ANOVA in standard form and Table 56 shows the Tukey’s pairwise results. The tibiotarsus Bp shows the groups are normally distributed (Shapiro-wilk p value of 0.207). The Levene’s test for homogeneity of variance reports a p value of 0.319 for the equality of variances, so there is no significant difference. This means all the assumptions of the ANOVA are safely met. Table 51 presents the results of the one-way ANOVA in standard form and Table 57 shows the Tukey’s pairwise results. The tibiotarsus SC shows the groups are normally distributed (Shapiro-wilk p value of 0.658). The Levene’s test for homogeneity of variance reports a p value of 0.406 for the equality of variances, so there is no significant difference. This means all the assumptions of the ANOVA are safely met. Table 52 presents the results of the one-way ANOVA in standard form and Table 58 shows the Tukey’s pairwise results. The tibiotarsus Bd shows the groups are normally distributed (Shapiro-wilk p value of 0.594). The Levene’s test for homogeneity of variance reports a p value of 0.986 for the equality of variances, so there is no significant difference. This means all the assumptions of the ANOVA are safely met. Table 53 presents the results of the one-way ANOVA in standard form and Table 59 shows the Tukey’s pairwise results. The tibiotarsus Dd measurement shows a relatively normal distribution when glancing at the residual graphs, however, Shapiro-wilk test for normality gives a p value of <.001. As the graphs show the data has a fairly normal distribution we continue to use the one-way ANOVA and do not move to a non-parametric test. One measurement of *F. a. grabae* is especially small and is likely to have caused the abnormality in the data. The Levene’s test for homogeneity reports a p value of <.001 for the equality of variances, this means that the variances are unequal, this compromises the assumptions of a one-way ANOVA. As a result Welch’s F test (in the case of unequal variances) was consulted and included in Table 54 along with the one-way ANOVA results, as the p value of the Welch’s test was significant (p=0.003) it was possible to continue to Tukey’s pairwise (Table 60).

| **Table 49**, Tibiotarsus La one-way ANOVA | | | | | |
| --- | --- | --- | --- | --- | --- |
|  | **Sum of sqrs** | **df** | **Mean square** | ***F*** | ***p*(same)** |
| **Between groups** | 301.08 | 2 | 150.54 | 36.56 | 3.133E-10 |
| **Within groups** | 189.417 | 46 | 4.11776 |  |  |
| **Welch *F* test** |  | 11.68 |  | 40.96 | 5.256E-06 |

One-way ANOVA results for the axial length (La) of the tibiotarsus. Taking into account only modern material.

| **Table 50**, Tibiotarsus Dip one-way ANOVA | | | | | |
| --- | --- | --- | --- | --- | --- |
|  | **Sum of sqrs** | **df** | **Mean square** | ***F*** | ***p*(same)** |
| **Between groups** | 5.11902 | 2 | 2.55951 | 17.96 | 1.848E-06 |
| **Within groups** | 6.41388 | 45 | 0.142531 |  |  |

One-way ANOVA results for the diagonal of the proximal end (Dip) of the tibiotarsus. Taking into account only modern material.

| **Table 51**, Tibiotarsus Bp one-way ANOVA | | | | | |
| --- | --- | --- | --- | --- | --- |
|  | **Sum of sqrs** | **df** | **Mean square** | ***F*** | ***p*(same)** |
| **Between groups** | 4.25193 | 2 | 2.12597 | 20.69 | 5.563E-07 |
| **Within groups** | 4.31503 | 42 | 0.102739 |  |  |

One-way ANOVA results for the breadth of the proximal end (Bp) of the tibiotarsus. Taking into account only modern material.

| **Table 52**, Tibiotarsus SC one-way ANOVA | | | | | |
| --- | --- | --- | --- | --- | --- |
|  | **Sum of sqrs** | **df** | **Mean square** | ***F*** | ***p*(same)** |
| **Between groups** | 0.852747 | 2 | 0.426374 | 8.222 | 0.0008852 |
| **Within groups** | 2.38536 | 46 | 0.0518557 |  |  |

One-way ANOVA results for the smallest breadth of the corpus (SC) of the tibiotarsus. Taking into account only modern material.

| **Table 53**, Tibiotarsus Bd one-way ANOVA | | | | | |
| --- | --- | --- | --- | --- | --- |
|  | **Sum of sqrs** | **df** | **Mean square** | ***F*** | ***p*(same)** |
| **Between groups** | 3.16184 | 2 | 1.58092 | 21.97 | 2.009E-07 |
| **Within groups** | 3.31033 | 46 | 0.0719636 |  |  |

One-way ANOVA results for the breadth of the distal end (Bd) of the tibiotarsus. Taking into account only modern material.

| **Table 54**, Tibiotarsus Dd one-way ANOVA | | | | | |
| --- | --- | --- | --- | --- | --- |
|  | **Sum of sqrs** | **df** | **Mean square** | ***F*** | ***p*(same)** |
| **Between groups** | 2.83447 | 2 | 1.41724 | 6.932 | 0.002337 |
| **Within groups** | 9.40488 | 46 | 0.204454 |  |  |
| **Welch *F* test** |  | 11.85 |  | 10.23 | 0.002622 |

One-way ANOVA results for the depth of the distal end (Dd) of the tibiotarsus. Taking into account only modern material.

| **Table 55**, Tibiotarsus La ANOVA - Tukey’s pairwise | | | |  | **Table 56**, Tibiotarsus Dip ANOVA - Tukey’s pairwise | | | |
| --- | --- | --- | --- | --- | --- | --- | --- | --- |
|  | ***F. a. grabae*** | ***F. a. arctica*** | ***F. a. naumanni*** |  |  | ***F. a. grabae*** | ***F. a. arctica*** | ***F. a. naumanni*** |
| ***F. a. grabae*** | - | 0.756 | <.001 |  | ***F. a. grabae*** | - | 0.002 | 0.997 |
| ***F. a. arctica*** | 1.011 | - | <.001 |  | ***F. a. arctica*** | 5.143 | - | <.001 |
| ***F. a. naumanni*** | 6.888 | 11.9 | - |  | ***F. a. naumanni*** | 0.097 | 7.778 | - |
|  |  |  |  |  |  |  |  |  |
|  |  |  |  |  |  |  |  |  |
| **Table 57**, Tibiotarsus Bp ANOVA - Tukey’s pairwise | | | |  | **Table 58**, Tibiotarsus SC ANOVA - Tukey’s pairwise | | | |
|  | ***F. a. grabae*** | ***F. a. arctica*** | ***F. a. naumanni*** |  |  | ***F. a. grabae*** | ***F. a. arctica*** | ***F. a. naumanni*** |
| ***F. a. grabae*** | - | 0.644 | <.001 |  | ***F. a. grabae*** | - | 0.759 | 0.009 |
| ***F. a. arctica*** | 1.272 | - | <.001 |  | ***F. a. arctica*** | 1.004 | - | 0.002 |
| ***F. a. naumanni*** | 6.114 | 8.505 | - |  | ***F. a. naumanni*** | 4.368 | 5.191 | - |
|  |  |  |  |  |  |  |  |  |
|  |  |  |  |  |  |  |  |  |
| **Table 59**, Tibiotarsus Bd ANOVA - Tukey’s pairwise | | | |  | **Table 60**, Tibiotarsus Dd ANOVA - Tukey’s pairwise | | | |
|  | ***F. a. grabae*** | ***F. a. arctica*** | ***F. a. naumanni*** |  |  | ***F. a. grabae*** | ***F. a. arctica*** | ***F. a. naumanni*** |
| ***F. a. grabae*** | - | 1 | <.001 |  | ***F. a. grabae*** | - | 0.108 | 0.002 |
| ***F. a. arctica*** | 0.039 | - | <.001 |  | ***F. a. arctica*** | 2.922 | - | 0.046 |
| ***F. a. naumanni*** | 5.935 | 9.061 | - |  | ***F. a. naumanni*** | 5.042 | 3.47 | - |

# Tarsometatarsus

The tarsometatarsus GL shows the groups are normally distributed (Shapiro-wilk p 0.277). The Levene’s test for homogeneity of variance reports a p value of 0.253 for the equality of variances, so there is no significant difference. This means all the assumptions of the ANOVA are safely met. Table 61 presents the results of the one-way ANOVA in standard form and Table 65 shows the Tukey’s pairwise results. The tarsometatarsus Bp shows the groups are normally distributed (Shapiro-wilk p 0.962). The Levene’s test for homogeneity of variance reports a p value of 0.741 for the equality of variances, so there is no significant difference. This means all the assumptions of the ANOVA are safely met. Table 62 presents the results of the one-way ANOVA in standard form and Table 66 shows the Tukey’s pairwise results. The tarsometatarsus SC shows the groups are normally distributed (Shapiro-wilk p value of 0.256). The Levene’s test for homogeneity of variance reports a p value of 0.05 for the equality of variances, this means that the variances are unequal, this compromises the assumptions of a one-way ANOVA. As a result Welch’s F test (in the case of unequal variances) was consulted and included in Table 63 along with the one-way ANOVA results, as the p value of the Welch’s test was significant (p=<.001) it was possible to continue to Tukey’s pairwise (Table 67). The tarsometatarsus Bd shows the groups are normally distributed (Shapiro-wilk p value of 0.653). The Levene’s test for homogeneity of variance reports a p value of 0.896 for the equality of variances, so there is no significant difference. This means all the assumptions of the ANOVA are safely met. Table 64 presents the results of the one-way ANOVA in standard form and Table 68 shows the Tukey’s pairwise results.

| **Table 61**, Tarsometatarsus GL one-way ANOVA | | | | | |
| --- | --- | --- | --- | --- | --- |
|  | **Sum of sqrs** | **df** | **Mean square** | ***F*** | ***p*(same)** |
| **Between groups** | 46.1549 | 2 | 23.0774 | 18.06 | 3.075E-06 |
| **Within groups** | 48.5663 | 38 | 1.27806 |  |  |

One-way ANOVA results for the greatest length (GL) of the tarsometatarsus. Taking into account only modern material.

| **Table 62**, Tarsometatarsus Bp one-way ANOVA | | | | | |
| --- | --- | --- | --- | --- | --- |
|  | **Sum of sqrs** | **df** | **Mean square** | ***F*** | ***p*(same)** |
| **Between groups** | 4.41039 | 2 | 2.20519 | 22.17 | 4.168E-07 |
| **Within groups** | 3.78029 | 38 | 0.0994813 |  |  |

One-way ANOVA results for the breadth of the proximal end (Bp) of the tarsometatarsus. Taking into account only modern material.

| **Table 63**, Tarsometatarsus SC one-way ANOVA | | | | | |
| --- | --- | --- | --- | --- | --- |
|  | **Sum of sqrs** | **df** | **Mean square** | ***F*** | ***p*(same)** |
| **Between groups** | 0.83093 | 2 | 0.415465 | 6.123 | 0.005045 |
| **Within groups** | 2.51051 | 37 | 0.0678515 |  |  |
| **Welch *F* test** |  | 23.55 |  | 26.64 | 8.982E-07 |

One-way ANOVA results for the smallest breadth of the corpus (SC)) of the tarsometatarsus. Taking into account only modern material.

| **Table 64**, Tarsometatarsus Bd one-way ANOVA | | | | | |
| --- | --- | --- | --- | --- | --- |
|  | **Sum of sqrs** | **df** | **Mean square** | ***F*** | ***p*(same)** |
| **Between groups** | 2.14758 | 2 | 1.07379 | 14.55 | 2.519E-05 |
| **Within groups** | 2.58299 | 35 | 0.0737997 |  |  |

One-way ANOVA results for the breadth of the distal end (Bd) of the tarsometatarsus. Taking into account only modern material.

| **Table 65**, Tarsometatarsus GL ANOVA - Tukey’s pairwise | | | |  | **Table 66**, Tarsometatarsus Bp ANOVA - Tukey’s pairwise | | | |
| --- | --- | --- | --- | --- | --- | --- | --- | --- |
|  | ***F. a. grabae*** | ***F. a. arctica*** | ***F. a. naumanni*** |  |  | ***F. a. grabae*** | ***F. a. arctica*** | ***F. a. naumanni*** |
| ***F. a. grabae*** | - | 0.028 | 0.897 |  | ***F. a. grabae*** | - | 0.700 | 0.003 |
| ***F. a. arctica*** | 3.804 | - | <.001 |  | ***F. a. arctica*** | 1.145 | - | <.001 |
| ***F. a. naumanni*** | 0.630 | 8.237 | - |  | ***F. a. naumanni*** | 5.059 | 9.01 | - |
|  |  |  |  |  |  |  |  |  |
|  |  |  |  |  |  |  |  |  |
| **Table 67**, Tarsometatarsus SC ANOVA - Tukey’s pairwise | | | |  | **Table 68**, Tarsometatarsus Bd ANOVA - Tukey’s pairwise | | | |
|  | ***F. a. grabae*** | ***F. a. arctica*** | ***F. a. naumanni*** |  |  | ***F. a. grabae*** | ***F. a. arctica*** | ***F. a. naumanni*** |
| ***F. a. grabae*** | - | 0.898 | 0.152 |  | ***F. a. grabae*** | - | 0.788 | 0.015 |
| ***F. a. arctica*** | 0.624 | - | 0.005 |  | ***F. a. arctica*** | 0.935 | - | <.001 |
| ***F. a. naumanni*** | 2.693 | 4.72 | - |  | ***F. a. naumanni*** | 4.177 | 7.255 | - |
