# Supplementary material for: Size variation in mid-Holocene North Atlantic Puffins indicates a dynamic response to climate change
Source: PLoS One. 2021 Feb 24;16(2):e0246888. doi: 10.1371/journal.pone.0246888 (PMC7904199; doi:10.1371/journal.pone.0246888)
Supplement: S1 Text — Description of all measurements featured within this paper. (DOCX) [file pone.0246888.s005.docx]

**S1 Text**. Measurements. Description of all measurements featured within the paper.

All measurements were taken following Von den Driesch (1976) or Kraft (1972). You will find the diagrams of the measurements on the pages indicated next to the description.

**Coracoid**

Medial length (Lm) – Similar to the greatest length measurement, however, measured from the processus acrocoracoideus to the angulus medialis. Diagram of the measurement can be found in Von den Driesch (1976, pp.113)

Basal breadth (Bb) – The distance from the processus lateralis to the angulus medialis. Diagram of the measurement can be found in Von den Driesch (1976, pp.113)

Breadth of the facies articularis sternalis (BF) – Measurement from the angulus medialis to the end of the facies articularis sternalis. Diagram of the measurement can be found in Von den Driesch (1976, pp.113)

**Humerus**

Greatest length (GL) – Measurement form the caput articulare humeri to the processus felxorious. Diagram of the measurement can be found in Von den Driesch (1976, pp.116)

Breadth of the proximal (Bp) – Breadth of the proximal end from the tuberculum laterale to the tuberculum mediale. Diagram of the measurement can be found in Von den Driesch (1976, pp.116)

Smallest breadth of the corpus (SC) – Measurement of the smallest breadth of the shaft, often around mid-shaft. Diagram of the measurement can be found in Von den Driesch (1976, pp.116)

Breadth of the distal end (Bd) – Measurement taken from the epicondylus medialis to the epicondylus lateralis. Diagram of the measurement can be found in Von den Driesch (1976, pp.116)

Smallest depth of the distal shaft – Measurement taken of the distal shaft above the condylus dorsalis. Diagram of the measurement can be found in Kraft (1972, pp.30)

**Ulna**

Greatest length (GL) – Measurement taken from the top of the olecranon to the conylus ventralis ulnae, giving the full length of the ulna. Diagram of the measurement can be found in Von den Driesch (1976, pp.118)

Diagonal of the proximal end (Dip) – Measurement of the greatest diagonal of the proximal end from the caudal border of the olecranon to the cranial border of the facies articularis. Diagram of the measurement can be found in Von den Driesch (1976, pp.118)

Breadth of the proximal end (Bp) – Measurement from the facies articularis medialis to the facies articularis lateralis. Diagram of the measurement can be found in Von den Driesch (1976, pp.118)

Depth of the proximal end (Tp) – Measurement from the top of the olecranon to the bottom of the cotyla dorsalis. Diagram of the measurement can be found in Kraft (1972, pp.32)

Smallest breadth of the corpus (SC) – Measurement of the smallest breadth of the shaft, often around mid-shaft. Diagram of the measurement can be found in Von den Driesch (1976, pp.118)

Diagonal of the distal end (Did) – Diagonal measurement from the tuberculum carpale to the condylus dorsalis ulnae. Diagram of the measurement can be found in Von den Driesch (1976, pp.118)

**Carpometacarpus**

Greatest length (GL) – Measurement taken from the trochlea carpalis to the most distal point, the facies articularis digitalis major. Diagram of the measurement can be found in Von den Driesch (1976, pp.120)

Breadth of the proximal end (Bp) – breadth of the proximal extremity, measurement taken from the tip of the processus extensorius to the edge of the facies articularis ulnocarpalis. Diagram of the measurement can be found in Von den Driesch (1976, pp.120)

Diagonal of the distal end (Did) – This measurement is of the distal articular surface only. Diagram of the measurement can be found in Von den Driesch (1976, pp.120)

Height of the symphysis (HS) – Measurement taken from the bottom of the spatium intermetacarpale to the facies articularis digitalis III. Diagram of the measurement can be found in Kraft (1972, pp.32)

**Femur**

Greatest length (GL) – Measurement taken from the top of the trochanter femoris to the furthest distal point, the condylus lateralis. Diagram of the measurement can be found in Von den Driesch (1976, pp.125)

Breadth of the proximal end (Bp) – Measurement taken from the caput femoris to the most lateral point of the trochanter major. Diagram of the measurement can be found in Von den Driesch (1976, pp.125)

Depth of the proximal end (Dp) – Measurement is taken from the fixed location of the cranial points on the caput femoris and on the trochanter major. Diagram of the measurement can be found in Von den Driesch (1976, pp.125)

Smallest breadth of the corpus (SC) – Measurement of the smallest breadth of the shaft, often around mid-shaft. Diagram of the measurement can be found in Von den Driesch (1976, pp.125)

Breadth of the distal end (Bd) – Measurement taken of the lateral and medial aspect of the condylus medialis and the condylus lateralis. Diagram of the measurement can be found in Von den Driesch (1976, pp.125)

Depth of the distal end (Dd) – Measurement taken from the fixed location of the caudal points of the condyli medialis and lateralis. Diagram of the measurement can be found in Von den Driesch (1976, pp.125)

**Tibiotarsus**

Axial length (La) – Measurement taken from the tuberculum centrale to the distal border of the trochlea tibiotarsi. Diagram of the measurement can be found in Von den Driesch (1976, pp.126–127)

Diagonal of the proximal end (Dip) – Measurement taken from the condylus medialis femoralis to the crista lateralis. Diagram of the measurement can be found in Von den Driesch (1976, pp.126–127)

Breadth of the proximal end (Bp) – Measurement taken from the edge of the facies articularis lateralis (where the caput fibulae is found) to the facies articularis medialis. Diagram of the measurement can be found in Kraft (1972, pp.34)

Smallest breadth of the corpus (SC) – Measurement of the smallest breadth of the shaft, often around mid-shaft. Diagram of the measurement can be found in Von den Driesch (1976, pp.126–127)

Breadth of the distal end (Bd) – Measurement taken of the lateral and medial aspect of the condylus medialis and the condylus lateralis. Diagram of the measurement can be found in Von den Driesch (1976, pp.126–127)

Depth of the distal end (Dd) – Measurement taken from the fixed points on the distal condyles taken from the caudal aspect. Diagram of the measurement can be found in Von den Driesch (1976, pp.126–127)

**Tarsometatarsus**

Greatest length (GL) – Measurement taken from the condylus interarticularis to the trochlea metatarsi III. Diagram of the measurement can be found in Von den Driesch (1976, pp.129)

Breadth of the proximal (Bp) – Measurement of the maximum breadth of the proximal end, taken from the facies articularis medialis to the facies articularis lateralis. Diagram of the measurement can be found in Von den Driesch (1976, pp.129)

Smallest breadth of the corpus (SC) – Measurement of the smallest breadth of the shaft, often around mid-shaft. Diagram of the measurement can be found in Von den Driesch (1976, pp.129)

Breadth of the distal end (Bd) – Measurement of the maximum breadth of the distal end from the trochlea metatarsi II to the trochlea metatarsi IV. Diagram of the measurement can be found in Von den Driesch (1976, pp.129)
